# Single-chain antibody gene therapy strategy based on high-throughput screening triggers sustained antiviral activity in the body

**DOI:** 10.1128/jvi.01497-24

**Published:** 2024-12-23

**Authors:** Liang Zhao, Xue-Feng Wei, Kun Xu, Zhao Zhao, Guo Chen, Hou-Peng Wang, Bin Zhu

**Affiliations:** 1 College of Animal Science and Technology, Northwest A&F University College of Animal Science and Technology 546344 , Yangling, China; 2 State Key Laboratory of Freshwater Ecology and Biotechnology, Hongshan Laboratory, Institute of Hydrobiology, Innovation Academy for Seed Design, Chinese Academy of Sciences 543760 , Wuhan, China; 3 Engineering Research Center of the Innovation and Development of Green Fishery Drugs, Universities of Shaanxi Province, Northwest A&F University 12469 , Yangling, China; St. Jude Children's Research Hospital, Memphis, Tennessee, USA

**Keywords:** viral diseases, single-chain antibodies, neutralization gene therapy, antibody gene transfer

## Abstract

The occurrence of viral diseases poses a huge threat and impact on human public health safety and the development of the animal and fishery industry. Here, a strain of single-chain antibody fragment, scFv-1, was isolated from the phage antibody display library construct by immunizing New Zealand white rabbits with rhabdovirus. *In vitro* analysis showed that the single-chain antibody could inhibit the infection of the virus in multiple pathways, including adsorption, fusion, and release. *In vivo* analysis revealed scFv-1 had a preventive and protective effect against the infection of virus. In addition, we describe that transposon-based transport of neutralizing genes allows for long-term, continuous expression, avoiding the need for lifelong, repeated passive immunization for treatment. In sum, high-throughput screening of neutralization genes based on phage display technology and transposon vector-based gene transfer provides effective methods for treating and preventing diseases and avoiding repetitive passive immunotherapy. This study also provides a reference for the prevention and treatment of unknown pathogens.

**IMPORTANCE:**

Livestock and fisheries play an important role in economic development and food security. The frequent outbreaks of viral diseases have caused great losses to the livestock industry, while the increase in drug resistance caused by the use of antibiotics as well as the potential risks to human health have raised serious concerns. Here, we constructed a phage display antibody library by immunizing New Zealand white rabbits with purified rhabdovirus and selected a single-chain antibody, scFv-1, with good neutralizing activity, which was validated and found to be able to block multiple phases of the virus and thus play a neutralizing role. In addition, we describe that transposon-based transport of neutralizing genes allows for long-term, continuous expression, reducing the need for lifelong, repeated passive immunization for treatment. Our work not only provides methods for the prevention and treatment of viral diseases but also provides the body with long-lasting and even permanent protection against repeated passive immunization.

## INTRODUCTION

Neutralizing antibodies (nAbs), which came to public attention and gained popularity in the scientific community as a result of the outbreak of coronavirus disease 2019, have partially accelerated the development of drugs and vaccines against unknown pathogens (1). Currently, there is a growing emphasis on the research and development of neutralizing antibodies against severe acute respiratory syndrome coronavirus 2 (SARS-CoV-2) (2–4). Besides SARS-CoV-2, nAbs have been widely used in the prophylaxis and treatment of various diseases, such as HIV, influenza, and malaria (5–7). Single-chain antibody fragment (scFv), a subset of neutralizing antibodies which contain an intact heavy-chain variable region and light-chain variable region, is the smallest fragment with complete antigen-binding activity and has attracted much attention from researchers because of the weak immunogenicity and low production costs (8, 9). Meanwhile, the mature technology system based on phage display greatly shortens the production and screening time of recombinant antibodies, making scFv’s a priority choice for biotechnology applications (10, 11). At present, scFv’s have been extensively used in the diagnosis and treatment of human and animal diseases, significantly promoting the advancements in medicine and agriculture (12–14).

Although passive infusion of nAbs can attenuate the onset of viral disease, it can still lead to disease over time and with viral re-infestation. Long-term, continuous, and systematic expression of the corresponding neutralizing antibodies in the body by a single administration may be a proven method of treating of the disease (15, 16). In recent years, the rapid development of biotechnology, especially the gene-editing technology, has provided new horizons for disease prevention and treatment. On the one hand, various bioreactor individuals, such as gene-edited chicken, rabbit, rat, sheep, and cow, were generated with antibody genes integrated and were proven to be capable to produce abundant antibodies (17–19). The antibody products were reported with retained immunological biological activity and can be used for large-scale applications in medical research and practice (20). Researchers find that a strain of anti-CD20 monoclonal antibody produced by a chicken bioreactor exhibited greater potential for treating cancer (21). On the other hand, the knock-in mouse models based on antibody heavy or light chain have been widely used in the design of immunogens against HIV, which have stimulated the specific immune responses and accelerated the development of HIV vaccines (22–24). From this, we hypothesize that individuals with specific antibody gene integrated will be able to fight the disease without any interventions when the corresponding pathogen invades and that their offspring would be able to mitigate the effects of the inherited disease.

Zebrafish, a widely used model organism in addition to mouse, which is characterized by rapid development, high fecundity, and large spawning capacity, can effectively improve the experimental efficiency (25). To test the above hypothesis, zebrafish was used as the living model in the current study. The spring viremia of carp virus (SVCV), a rhabdovirus with worldwide economic and environmental importance, was used as the model virus (26). Currently, although a large number of drug candidates, including arctigenin, anisomycin, and bavamycin, have been reported to be effective in inhibiting the replication of SVCV, the emergence of neutralizing antibodies against SVCV has rarely been reported (27–29). First, the New Zealand white rabbits were immunized with the purified SVCV, and a phage antibody display library was constructed. Subsequently, single-chain antibodies with high specificity were screened by phage display technology, and the ability of the antibodies to neutralize the SVCV *in vitro* was evaluated, while computer simulation technology was used to further locate the identified antigenic sites which were verified by alanine mutation. In addition, its application prospect was explored though evaluating the preventive and therapeutic effects *in vivo*. Finally, zebrafish with constitutively expressing neutralizing antibody were obtained based on transposon vector delivery of neutralizing genes, and the therapeutic effect was evaluated to validate the concept.

## RESULTS

### The serum from immunized New Zealand white rabbits neutralizes SVCV

**Fig 1:**
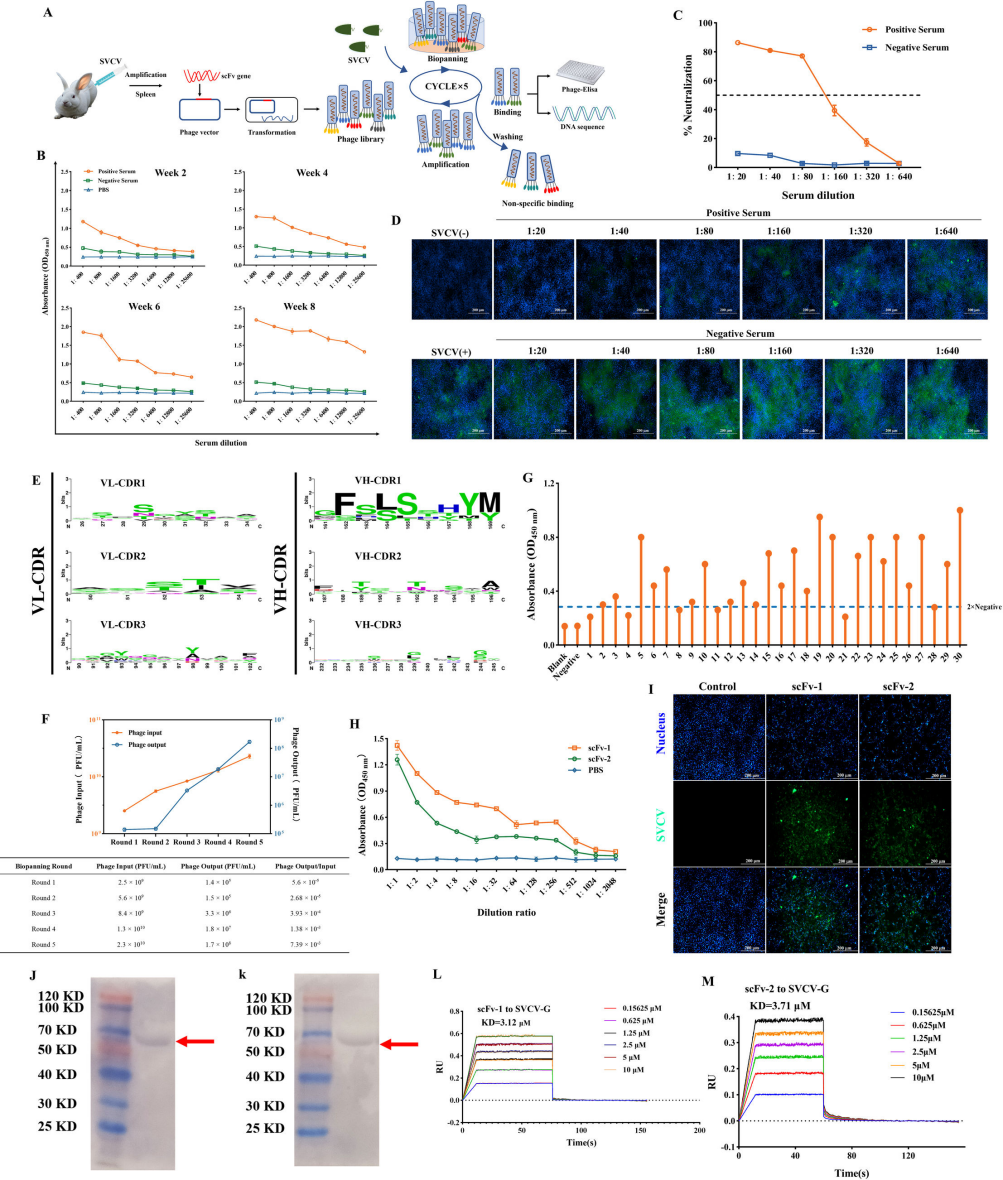
Construction of anti-SVCV phage library and the biopanning and identification of single-chain antibody. (**A**) Schematic diagram of the biopanning strategy for anti-SVCV-specific scFv’s. The purified SVCV was used as the target, and the immunopositive phage clones were further identified through sequencing and enzyme-linked immunosorbent assay (ELISA) after five rounds of biopanning. (**B**) The titers of polyclonal antibodies in serum at different time points after immunization were analyzed using ELISA. (**C**) The neutralization effect of serum samples on SVCV at the eighth week after immunization was evaluated by the MTT assay. The serum was diluted from 1:20 to 1:640 in a twofold ratio, and the serum samples were re-tested three times. Results are presented as mean ± SD. (**D**) Indirect immunofluorescence assay was used to detect the neutralizing effect of serum on SVCV, which increased with the increase in serum concentration. Blue indicates the nucleus; green denotes SVCV. Scale: 200 µm. (**E**) Diversity analysis of the constructed SVCV immune rabbit phage display single-chain antibody library was performed by randomly selecting multiple single colonies for sequencing analysis. (**F**) Monitoring of the output:input ratio of phages in five consecutive rounds of biopanning. (**G**) Randomly select 30 positive phage plaques from the phage library after five rounds of biological selection for ELISA detection. (**H**) Affinity of purified scFv’s to SVCV detected by indirect ELISA. Results are presented as mean ± SD. (**I**) Indirect immunofluorescence assay was used to detect the specific reaction between scFv’s and SVCV in infected cells. Blue indicates the nucleus; green denotes SVCV. Scale: 200 µm. (**J and K**) The ability of the scFv’s to bind to each of the SVCV proteins by Western blot assay; samples were prepared using purified virus particles. (**L and M**) The affinity of the scFv’s for SVCV-G protein was detected by biolayer interferometry assay.

New Zealand white rabbits were immunized with purified SVCV particles, and high-titer serum antibodies with specific neutralizing effect against SVCV were induced and detected. As shown in Fig. 1A, healthy young New Zealand white rabbits were immunized four rounds by subcutaneous injection. The SVCV elicited robust immunity, and the serum antibody titer increased significantly with the increase of immunization times (Fig. 1B). After immunization, the antibody titer reached a peak of 1:256,00 at week 8, which was significantly higher than that of the negative control group (Fig. S1A). Besides, the immunofluorescence (IF) assay demonstrated that the positive sera reacted with the SVCV-infected cells, whereas the negative control sera and phosphate-buffered saline (PBS) did not, suggesting that specific antibodies against SVCV were successfully produced by the immunized rabbits (Fig. S1B). Furthermore, to assess the neutralizing activity of the antiserum, the reactivity of the serum against SVCV was examined by MTT [3-(4,5)-dimethylthiazo(lz-yl-3,5-phenytetrazoliumromide)]. The results showed the antiserum was effective in neutralizing SVCV with an IC_50_ (half-maximal inhibitory value of activity, expressed as the reciprocal of the serum with 50% neutralizing activity) of 153.71 (Fig. 1C). Similarly, co-incubation of the virus with the serum followed by the infection of epithelioma papulosum cyprinid (EPC) cells showed that the infectivity of the virus decreased significantly with increased serum concentration (Fig. 1D). Overall, these results indicated that the specific antibodies can be produced by SVCV-immunized rabbits to neutralize SVCV.

### Screening anti-SVCV single-chain antibodies by phage display technology

To obtain the anti-SVCV single-chain antibodies (scFv), we constructed a phage display scFv library based on the SVCV-immunized rabbit spleen B cells. As shown in Fig. S2A and B, the V_H_ and V_L_ genes were amplified using the reverse-transcribed cDNA as the template, and the fragment sizes were 384 and 366 bp, respectively. Then, the long complementary linker sequences were added and amplified, and the purified VL-linker and linker-VH were spliced into the whole *scFv* gene by splicing overlap extension PCR (SOE-PCR), where the fragment size was 795 bp (Fig. S2C). After double digestion with *Sfi* I and *Not* I, the pCANTAB 5E-scFv recombinant phage-plasmids were transformed into TG1 to construct an SVCV-immunized rabbit-derived phage display scFv library with a library capacity of 2.1 × 10^5^ CFU/mL. The randomly selected single colony (scFv gene) was detected by PCR, and the results showed the positive recombination rate was 95% (Fig. S2E). In addition, all those *scFv* genes were found possessing a rich diversity at V_L_ (CDR1-3) and V_H_ (CDR1-3) through sequence alignment (Fig. 1E).

Subsequently, five consecutive rounds of bioelution were performed using SVCV as bait, and the results showed the number of enriched phages increased from 1.4 × 10^5^ in the first round of panning to 1.7 × 10^8^ in the fifth round of biopanning, and the enrichment efficiency increased from 5.6 × 10^−5^ to 7.39 × 10^−3^(Fig. 1F). The phages specifically bound to the virus were effectively enriched after five rounds of panning, and the enrichment factor was 132-fold. After the fifth round of enrichment, 30 positive clones were randomly selected to determine the binding property to SVCV using phage enzyme-linked immunosorbent assay (ELISA). As shown in Fig. 1G, among them, 23 phage clones showed positive reactions (the optical density at 450 nm was 2.1 times higher than that of the negative group). Then, 10 positive clones with the highest affinity were selected for sequencing analysis. The results showed that 9 of the 10 positive clones had the same amino acid sequence, which was named scFv-1, and another was named scFv-2 (Fig. S3). At the same time, sequence alignment analysis showed there were significant differences between the two scFv’s in the variable region.

To prove that the binding of scFv antibodies with SVCV is independent of the phage particles, the *scFv* genes were subcloned into pET-32a and the proteins were prepared by prokaryotic expression in *Escherichia coli* BL21. SDS-PAGE and Western blotting were used to validate the purified scFv proteins, which were expressed correctly (Fig. S4). In addition, the affinity of scFv’s for SVCV was evaluated by ELISA, and the results showed that both scFv-1 and scFv-2 could bind to the virus effectively, and the binding ability of scFv-1 was better than that of scFv-2 (Fig. 1H). Furthermore, to evaluate whether the scFv’s had the ability to specifically recognize SVCV, the EPC cells were infected with SVCV for 48 h, and the scFv’s were used for immunofluorescence detection. As shown in Fig. 1I, both scFv-1 and scFv-2 reacted with infected EPC cells, and the specific fluorescence signal with respect to SVCV appeared mainly in the cytoplasm rather than in the nucleus. Furthermore, the binding ability of the antibody to each protein of SVCV was investigated using Western blot (WB) assay. It was found that both scFv’s bind only to the SVCV-G protein (Fig. 1J and K). Following this, the SVCV-G protein was purified, and the affinity of the scFv’s to the SVCV-G protein was detected using biolayer interferometry (BLI) assays (Fig. 1L and M). It is noteworthy that neither strain of scFv’s has a particularly high affinity for the SVCV-G protein, mainly due to the lack of the corresponding Fab fragment as well as the Fc fragment. In summary, the above results showed the scFv’s screened by phage display could bind to SVCV specifically.

### The single-chain antibodies scFv-1 and scFv-2 neutralize SVCV *in vitro*

**Fig 2:**
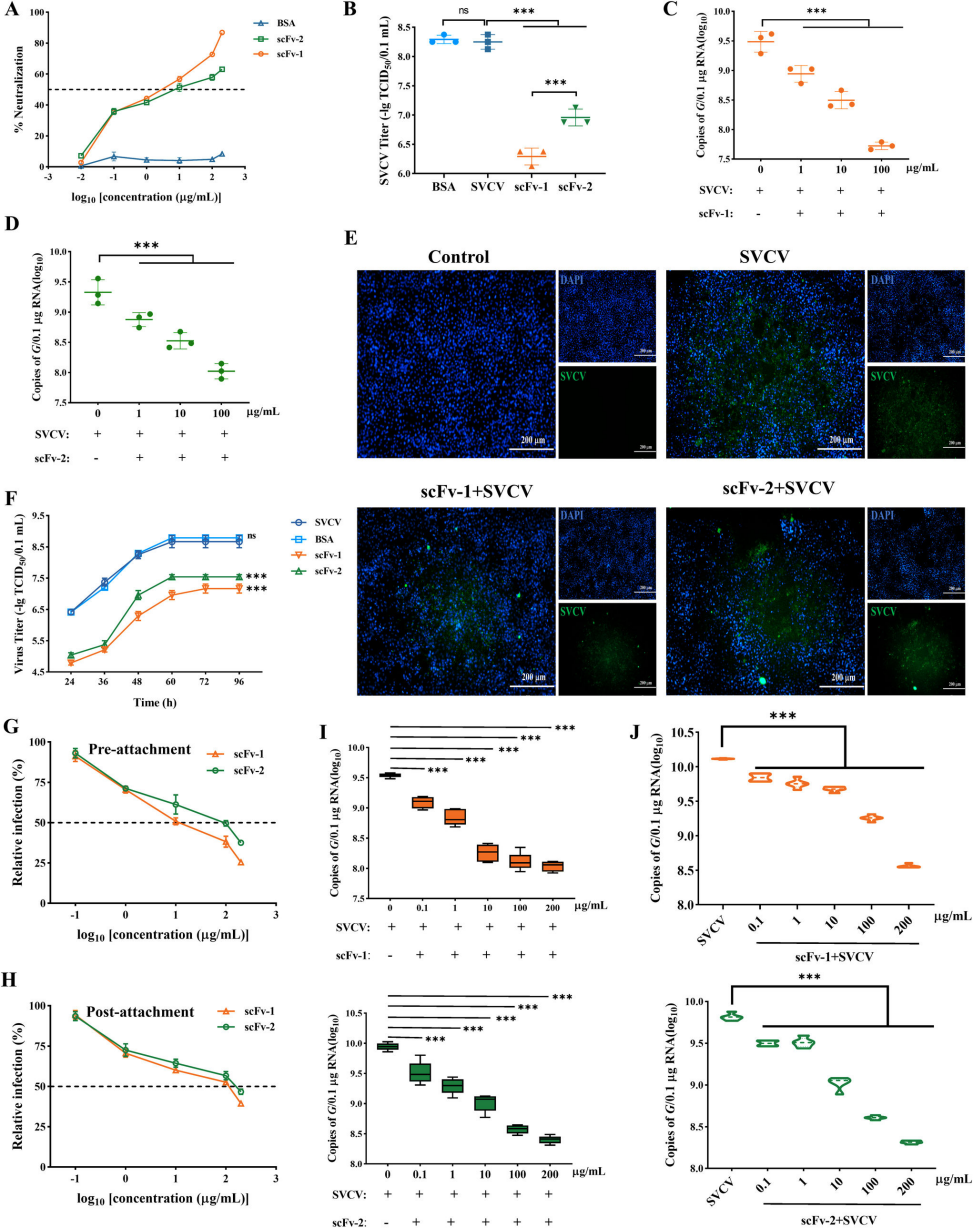
Neutralization characteristics of anti-SVCV-specific scFv’s *in vitro*. (**A**) The neutralization effect of scFv’s on SVCV was evaluated by the MTT method. Results are presented as mean ± SD. (**B**) Titer analysis of virus after scFv treatment. The viral load was evaluated by real-time quantitative PCR and expressed as copies of the SVCV *G* gene. Values are presented as mean ± SD. The viral load of cells infected with SVCV was detected after scFv-1 (**C**) and scFv-2 (**D**) treatments, respectively. ns, no significance, ***P < 0.001. (**E**) The neutralization effect of scFv on SVCV was detected using indirect immunofluorescence assay. The nucleus was stained with DAPI (4',6-diamidino-2-phenylindole, blue fluorescence), and the SVCV showed green fluorescence. Scale: 200 µm. (**F**) The purified scFv protein with a final concentration of 100 µg/mL was pre-incubated with SVCV at 25°C for 1 h, and then co-incubated with cells. The supernatant was collected at 24, 36, 48, 60, 72, and 96 h to determine TCID_50_. The values at different time points were used to reflect the growth curve of SVCV. (**G**) The scFv’s were pre-incubated with SVCV at 4°C for 1 h, followed by incubation of the antibody-virus mixture with EPC cells at 4°C for 1 h. After removal of non-adsorbed virus, the culture was maintained for 48 h, and the neutralization ability was assessed by MTT assay. (**H**) The EPC cells were co-incubated with SVCV at 4°C for 1 h, followed by addition of the scFv’s for 1 h. Subsequently, the medium was replaced with M199 containing 2% FBS, and the culture was continued for 48 h; the neutralization ability was assessed by MTT assay. The viral load was evaluated by RT-qPCR to reflect the ability of the scFv-1 (**I**) and scFv-2 (**J**) to neutralize SVCV at pre-attachment or post-attachment stage. Values are presented as mean ± SD. ***P < 0.001.

For the scFv antibodies obtained, the neutralizing activity to SVCV was assessed to determine their application potential. SVCV was incubated with different concentration of scFv-1 and scFv-2 to infect EPC cells, and the same concentration of bovine serum albumin (BSA) was incubated with SVCV as controls. As determined by MTT assay, both scFv-1 and scFv-2 improved the cell viability caused by SVCV infection in a dose-dependent manner. It is worth mentioning that the highest neutralization rate of scFv-1 and scFv-2 reached 86.85% and 63.12%, respectively (Fig. 2A). Similarly, SVCV was incubated with scFv-1 (100 µg/mL) and scFv-2 (100 µg/mL), respectively, to infect EPC cells, and then the supernatant was collected for virus titer detection. After scFv treatment, the virus titers were reduced from 10^8.25^ TCID_50_/mL to 10^6.25^ TCID_50_/mL and 10^6.95^ TCID_50_/mL, respectively (Fig. 2B).

To further evaluate the neutralizing potential of the scFv’s, we established a standard curve to quantify the *G* gene of SVCV for subsequent experiments (Fig. S5D). EPC cells were infected with SVCV that was pre-incubated with different concentrations of scFv-1 and scFv-2, and then the viral load was detected. The viral burden decreased significantly in a dose-dependent manner after scFv-1 pre-treatment, and the viral burden decreased by 4.43-, 11.58-, and 63.8-fold, respectively (Fig. 2C). Similarly, the viral burden correspondingly declined by 2.21-, 5.71-, and 15.79-fold after scFv-2 pre-treatment (Fig. 2D). To extend these findings, we detected the virus growth curve after scFv pre-treatment. The viral load was significantly lower after scFv treatment and increased at a slower rate with the extension of time, but the viral burden was less than that of the control group. In addition, the viral load after scFv-1 treatment was significantly lower than that of scFv-2 treatment group (Fig. 2F). In addition, the indirect immunofluorescence results showed that the fluorescence intensity after scFv treatment was significantly weaker than that of the control group infected with SVCV without any treatment (Fig. 2E, upper right corner). Meanwhile, we analyzed the apoptosis of SVCV-infected cells after scFv treatment. After SVCV infection, the number of apoptotic and necrotic cells significantly increased, while the number of apoptotic cells significantly decreased after scFv treatment, and no necrotic cells were observed (Fig. S6). In summary, the above results demonstrated that scFv-1 and scFv-2 were potently candidates for neutralizing SVCV.

Furthermore, we carried out pre-attachment and post-attachment neutralization assays. In the experiments, the scFv’s were added before or after the virus attachment. As shown in Fig. 2G and H, the scFv’s effectively decreased the infection ability of SVCV both before or after the virus absorbed to the cells. To extend these results, we detected the viral burden. As shown in Fig. 2I, both scFv-1 and scFv-2 neutralized the virus in a dose-dependent manner, and the viral burden was reduced by 77.24- and 35.03-fold, respectively, compared with the control group. When scFv’s were added after the virus attachment, the virus load was only reduced by 35.93- and 20.68-fold even at the highest scFv concentration (Fig. 2J). It was undeniable that the post-addition of scFv’s did reduce the viral burden, but the neutralization effect was not as satisfactory as expected. The reason was likely that when the virus bound to the cell surface, some areas in contact with the cells became less likely to be recognized by the antibodies, which led to the weakening of antibody neutralization (30). In sum, these results indicated the scFv’s could neutralize SVCV in the pre-attachment and post-attachment stages.

### The scFv-1 blocks multiple pathways in the viral infection cycle to exert the neutralizing effect

**Fig 3:**
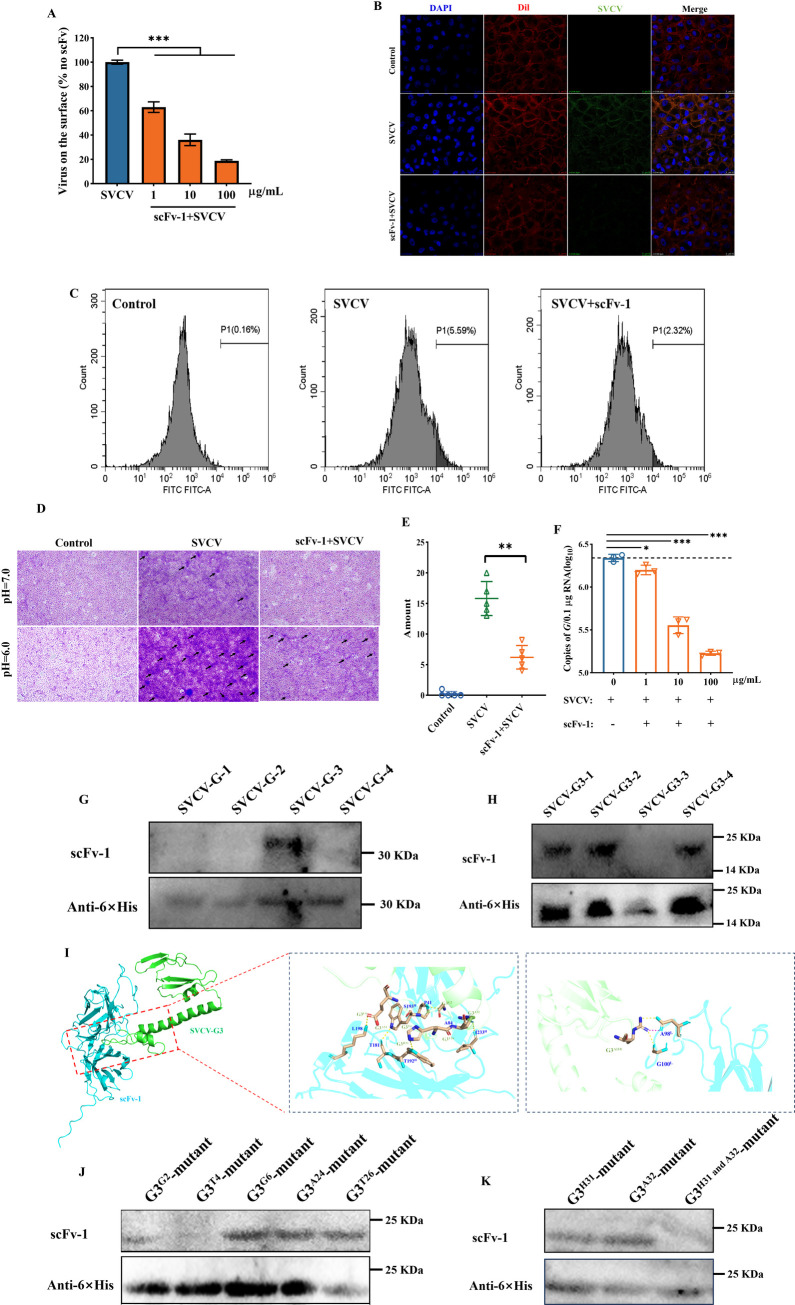
Evaluation of neutralization mechanism of the scFv-1. (**A**) The scFv-1 was pre-incubated with SVCV at 4°C for 1 h, and then the antibody-virus mixture was incubated with EPC cells at 4°C for 2 h. Subsequently, the cells were washed to remove the non-adsorbed virus, and the viral load on the cell surface was detected using RT-qPCR. Results are presented as mean ± SD. *P < 0.05, ***P < 0.001. (**B**) Virus on the cell surface was detected by indirect immunofluorescence using confocal microscopy. Red indicates the cell membrane; blue indicates the nucleus; green indicates the SVCV. Oil mirror, ×63. (**C**) Detection of fluorescence intensity of viral particles on the cell surface by flow cytometry. (**D**) The fusion of virus with cell membrane was inhibited by scFv-1 under acidic conditions. (**E**) Quantitative analysis of the number of syncytia. (**F**) The EPC cells were co-incubated with SVCV at 25°C for 2 h, and then scFv-1 was added to the co-incubation for 18 h. Subsequently, the supernatant was collected, and the viral load was detected by RT-qPCR. Results are presented as mean ± SD. *P < 0.05, ***P < 0.001. (**G**) The SVCV-G protein was divided into four segments using the truncated segmentation method, and the binding ability of the scFv-1 to them was detected using the WB assay after induced expression. (**H**) The SVCV-G3 protein was divided into four segments using the truncated segmentation method, and the binding ability of scFv-1 to these segments was detected using the WB assay after induced expression. (**I**) Details of interaction between scFv-1 and SVCV-G3. The right panel shows the hydrogen bound at the binding interface between scFv-1 (blue) and SVCV-G3 (green). (**J and K**) The amino acid sites at which the scFv-1 binds to the SVCV-G3 were sequentially targeted for mutation, and the ability of the scFv-1 to bind to the mutant was detected using the WB assay.

Due to the better neutralization effect *in vitro*, scFv-1 was selected for subsequent experiments. To explore the mechanism for the neutralization, we detected the roles that scFv-1 played in different stages of the viral infection. Primarily, we examined whether the SVCV and cell binding could be inhibited by scFv-1. The SVCV was co-incubated with scFv-1 before adding to the EPC cells at 4°C, a temperature condition that was supposed to prevent virus internalization. Then, the virus adsorbed to the cells were detected by real-time quantitative PCR (RT-qPCR). After the pre-incubation with scFv-1, the virus absorption was significantly inhibited, and the viral burden decreased significantly with the increased concentration of scFv-1 (Fig. 3A). To corroborate the finding, we next performed IF assay and flow cytometry to detect the virus and cell bind. As shown in Fig. 3B, the virus (green fluorescence) mainly existed on the surface of the cell membrane (red fluorescence), indicating the virus was adsorbed to the cells. After pre-incubation with the scFv-1, the fluorescence intensity was significantly weakened (Fig. 3C), which proved that scFv-1 inhibited the adsorption of the SVCV.

In addition to the virus entry, we detected if scFv-1 prevented the virus from endosomal membrane fusion and from penetrating into the cytoplasm. After adding the cell fusion medium, the virus produced obvious fusion phenomenon. A large number of aggregated multi-cells were observed after crystalline violet staining, and the addition of scFv-1 alleviated the fusion between the virus and cells to a certain extent (Fig. 3D and E). Furthermore, we evaluated if the egress of virus particles could be blocked by scFv-1. We detected the amount of the virus in the supernatant after the infection at different time points. The amount of SVCV in the supernatant increased significantly 18 h after the cells were infected (Fig. S7A), which was considered as the start of the first round of the virus secretion in this study. Based on this, we co-incubated the cells with SVCV for 2 h at 25°C and then removed the unbound virus by washing with PBS. Subsequently, the scFv-1 was co-incubated with infected EPC cells for 2, 10, and 18 h, and the amount of the virus in supernatant was detected. The results demonstrated scFv-1 significantly inhibited the viral egress at 18 h post-infection (Fig. 3F). However, as expected, scFv-1 did not completely inhibit the egress of SVCV. At the early time points (2 and 10 h) after the viral infection, the inhibitory effect of scFv-1 was not detected (Fig. S7B and C). We then co-incubated the scFv-1 with the EPC cells for 18 h and found that the scFv-1 could bind to the cell membrane, suggesting to some extent that the antibody inhibits the release of the SVCV by binding to certain sites on the cell membrane. However, the viral particles were still released in large numbers over time, thus suggesting that the antibodies inhibited only the release of early viral particles (Fig. S8). In sum, scFv-1 neutralized SVCV through the inhibition of the viral adsorption, fusion inhibition, and viral egress.

### Structural localization of the antigenic regions recognized by scFv-1

As the major protein on the surface of rhabdovirus, G protein mediated viral endocytosis by binding to receptors on the surface of target cells and subsequently inducing fusion of the viral vesicle membrane with the endosomal membrane (31, 32). Most of the sites on the G proteins were not essential for recognition by the neutralizing antibody, so we truncated the expression of the SVCV-G protein and named them SVCV-G1,SVCV-G2,SVCV-G3, and SVCV-G4, respectively. Then, we evaluated the binding capacity of the antibody to each segment through Western blot analysis following the induced expression of each segment. The results showed that the scFv-1 exhibited binding affinity toward the SVCV-G3 segment (Fig. 3G). Subsequently, further truncation of the SVCV-G3 fragment was conducted, resulting in the discovery that the single-chain antibody exhibited binding capabilities to multiple segments (Fig. 3H). This result prompted us to conclude that the site identified by scFv-1 is not situated on a contiguous segment. Consequently, we employed AlphaFold3 Server for prediction and molecular docking for the SVCV-G3 protein and scFv-1, respectively. the results showed that the amino acid sites recognized by the antibody were Gly2, Trp4, Glu6, Asp24, Thr26, His31, Arg32, and Arg118 of SVCV-G3 (Fig. 3I). These predicted regions of amino acid site distribution basically matched with our preliminary WB assay results, which provided evidence of homology modeling and molecular docking. Meanwhile, we found that the antibody predominantly recognized amino acid residues between 1 and 60 of the SVCV-G3 fragment. In accordance with the distribution of the predicted sites, we truncated amino acid sites 1–60 of the SVCV-G3 fragment, comprising 30 amino acid sequences in each group, designated G3^1–30^ and G3^31–60^ (G3^1–30^ represented amino acids 1–30, containing the SVCV-G3 fragment, and G3^31–60^ represented amino acids 31–60, containing the SVCV-G3 fragment), respectively. Then. the amino acid sites were sequentially fixed-point mutated and combined with the WB assay; we found that the sites recognized by scFv-1 were mainly Trp4, His31, Arg32, and Arg118 of SVCV-G3 (Fig. 3J and K).

### Preventive and therapeutic administration of scFv-1 protect against SVCV challenge *in vivo*

**Fig 4:**
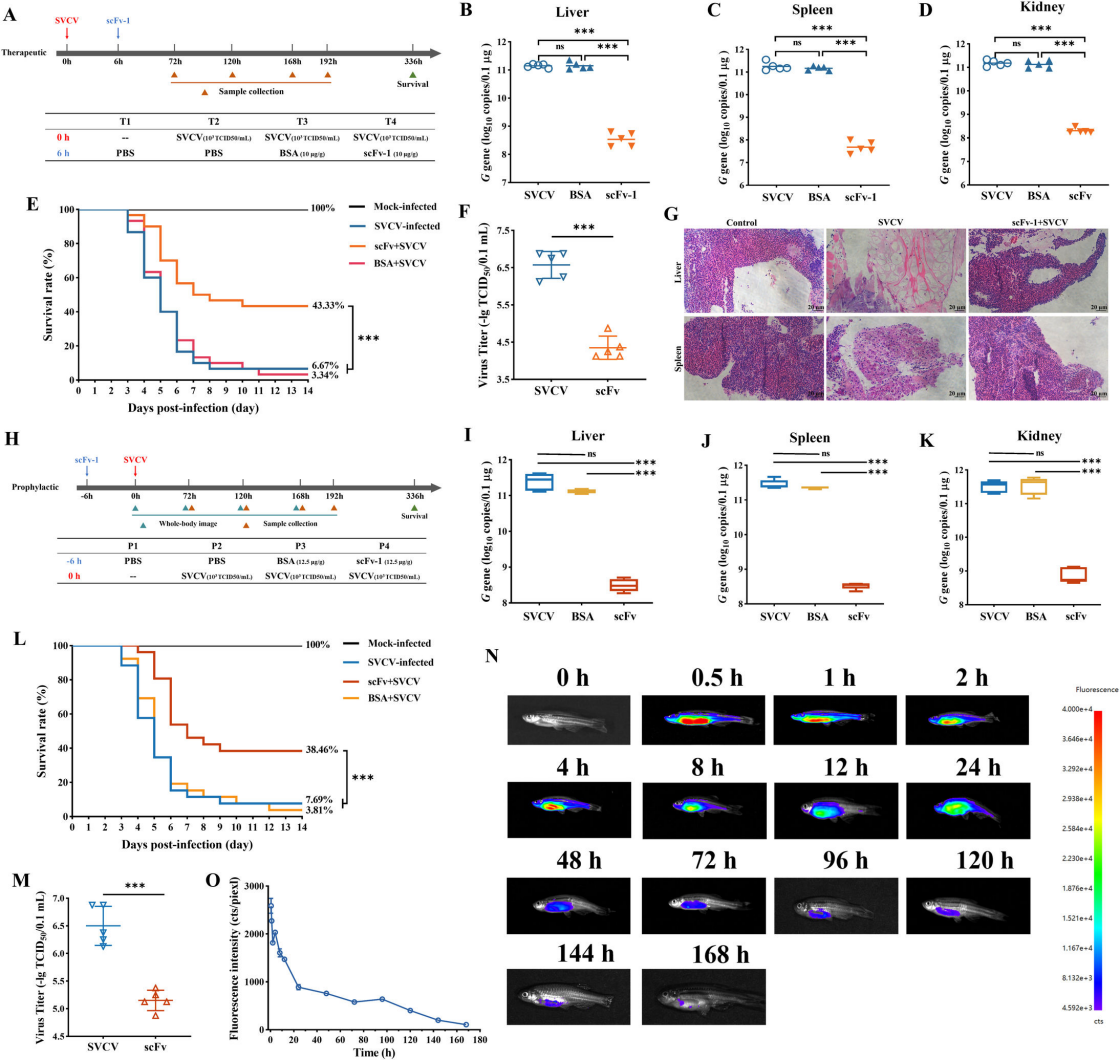
Prophylactic and therapeutic effect of scFv-1 against SVCV infection *in vivo*. (**A**) Schematic diagram of challenge and therapeutic strategy. Zebrafish were treated with the scFv-1 (concentration of 10 mg/kg) after intraperitoneal injection of SVCV virus for 6 h. The viral load was evaluated by RT-qPCR and expressed as copies of the SVCV *G* gene. Values are presented as mean ± SD. (B–D) The viral load in the liver, spleen, and kidney of SVCV-infected zebrafish on day 7 after scFv-1 treatment. ns, no sigficance, ***P < 0.001 (*n* = 5). (**E**) Survival rate of SVCV-infected fish within 14 days after scfv-1 treatment; the survival curve was calculated by GraphPad Prism software. (**F**) The virus titer analysis of zebrafish after scFv-1 treatment (*n* = 5). Data are presented as mean ± SD. ***P < 0.001. (**G**) After scFv-1 treatment, hematoxylin-eosin staining was used to observe pathological changes in liver and spleen tissues. (**H**) Schematic diagram of challenge and prophylactic strategy. Zebrafish were challenged with SVCV after intraperitoneal injection of scFv-1 (concentration of 12.5 mg/kg) for 6 h. (I–K) The viral load in the liver, spleen, and kidney of SVCV-infected zebrafish on day 7 after scFv-1 pre-treatment (*n* = 5). ns, no significance, ***P < 0.001. (**L**) Kaplan-Meier survival curve of SVCV-infected fish after scFv-1 pre-treatment within 14 days. (**M**) The virus titer analysis of zebrafish after scFv-1 treatment (*n* = 5). Data are presented as mean ± SD. ***P < 0.001. (N and O) Distribution and metabolic analysis at different time points after intraperitoneal injection of fluorescein isothiocyanate (FITC)-labeled scFv-1 in zebrafish (*n* = 3).

To further evaluate the neutralization ability of scFv-1, its prophylactic and therapeutic efficacy in SVCV-infected zebrafish models was tested. In order to detect the therapeutic effect against SVCV challenge, zebrafish were injected with SVCV (10^3^ TCID_50_/mL) intraperitoneally. After 6 h, scFv-1 with the dose of 10 mg/kg was administered by intraperitoneal injection (Fig. 4A). The survival rate of zebrafish without scFv-1 treatment was only 6.67% within 14 days, while the survival rate of zebrafish treated with scFv-1 after SVCV infection was significantly improved by 43.33% (Fig. 4E). After scFv-1 treatment, the viral burden in the liver, spleen, and kidney of the SVCV-infected zebrafish showed different degrees of decline. The viral burden was reduced by 412-, 3,671-, and 631-fold compared with the control group without scFv-1 treatment, respectively, on the seventh day after the virus infection (Fig. 4B through D). In addition, the virus growth curves in the liver, spleen, and kidney also showed the same results. After scFv-1 treatment, the viral burden content increased at a slower rate with time, which was consistent with the declined viral burden in multiple different tissues. However, the treatment with BSA had no significant effect on the virus replication (Fig. S11). Similarly, the titer detection also showed a significant decrease after scFv-1 treatment, and the titer declined from 10^6.75^ TCID_50_/mL to 10^4.375^ TCID_50_/mL (Fig. 4F). To corroborate these findings, the pathological analysis was further performed. The liver and spleen tissues of zebrafish in the SVCV infection group showed obvious pathological changes, including hemorrhage, vacuolization, and inflammatory cell infiltration, while the pathological changes were significantly alleviated after scFv-1 treatment (Fig. 4G).

For the prophylactic experiments, zebrafish were treated with scFv-1 (12.5 mg/kg) 6 h before being challenged with SVCV (10^3^ TCID_50_/mL) (Fig. 4H). The mortality rate of zebrafish in the non-antibody-treated group reached 92.31% within 14 days (the designated end point of this study). In contrast, the survival rate of zebrafish pre-treated with scFv-1 significantly improved by 38.46%. Notably, there was a significant lag in the death time of zebrafish after pre-treatment with scFv-1 (Fig. 4L). To extend these findings, we examined the effects of scFv-1 pre-treatment on viral burden in the liver, spleen, and kidney at days 3, 5, and 7 post-infection, respectively. In the spleen, the viral RNA levels showed different degrees of decline after scFv-1 pre-treatment on the third, fifth, and seventh days compared with the control group, which were reduced by about 174-, 348-, and 848-fold, respectively (Fig. 4J; Fig. S12). In addition, the viral burden increased over time but was significantly lower than that of the control group. In the same way, the viral burden in the liver and kidney also showed similar changes. The greatest decline in viral burden in the liver (~776-fold) and kidney (~434-fold) on the seventh day after the SVCV infection was observed in fish pre-treated with scFv-1 (Fig. 4I–K). It is worth noting that the viral burden in the liver, spleen, and kidney increased at a relatively slow rate compared with the control group, which also confirmed the lag of death time. We also measured the viral titer in SVCV-infected zebrafish. The titer decreased from 10^6.5^ TCID_50_/mL to 10^5.15^ TCID_50_/mL after scFv-1 pre-treatment (Fig. 4M), indicating that scFv-1 could play a preventive role in the SVCV infection. Furthermore, to assess the metabolism of scFv-1 *in vivo*, we injected scFv-1 intraperitoneally into zebrafish and collected the zebrafish for fluorescence imaging at different time points after injection. After a single injection, scFv-1 (labeled with FITC) was enriched in different tissues and persisted for 168 h in whole-body images (Fig. 4N and O). Also to exclude the effect of bacterially expressed proteins containing endotoxin on the results of the experiments, a strain of negative, non-specifically binding single-chain antibody to SVCV (purified using the same purification method as scFv-1) was subjected to the same experiments separately, and the results showed that the non-specific antibody was unable to neutralize SVCV (Fig. S9 and S10). Overall, scFv-1 could inhibit viral replication, reduce the pathological changes in liver and spleen, and exhibit efficient prophylactic and therapeutic efficacy against SVCV infection *in vivo*.

### Construction of zebrafish expressing scFv-1 and evaluation of therapeutic effects

**Fig 5:**
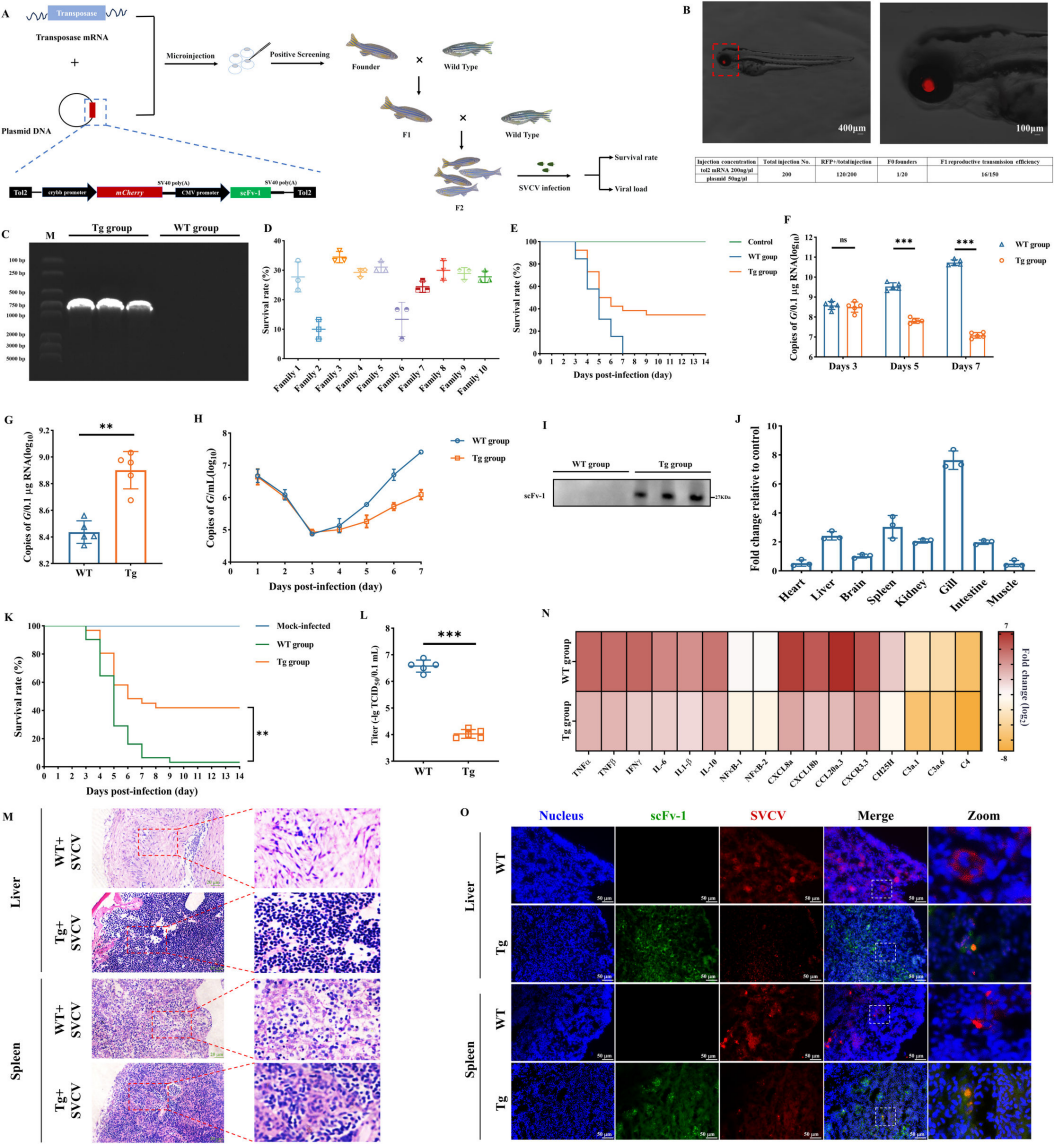
Construction of zebrafish expressing scFv-1 and evaluation of therapeutic effects. (**A**) Schematic diagram of the construction strategy for zebrafish with constitutively expressing neutralizing antibody. (**B**) Positive individuals were screened through fluorescence microscopy observation, and the eyes of the positive individuals were red due to the expression of mCherry protein. The enlarged details of the eyes are shown in the image on the right. Scale: 200 µm. (**C**) Zebrafish were randomly selected from F_2_ generation-positive individuals for PCR amplification of the *scFv-1* gene, and the amplification products were verified by agarose electrophoresis. Randomly select wild zebrafish were used as a control. (**D**) Survival rate of F_2_ generation-positive zebrafish individuals from different families within 14 days after infection with SVCV; the survival curve was calculated by GraphPad Prism software. (**E**) The survival rate of zebrafish in the Tg and WT groups after SVCV immersion infection within 14 days; the survival curve was calculated by GraphPad Prism software. The viral load was evaluated by RT-qPCR and expressed as copies of the SVCV *G* gene. Results are presented as mean ± SD. (**F**) The viral load in the zebrafish of the Tg group and the WT group after infection with SVCV on days 3, 5, and 7.ns, no significance, ***P < 0.001. (**G**) The viral load of dying zebrafish in the Tg group and the WT group after infection with SVCV. **P < 0.01. (**H**) Aquaculture water samples were collected for seven consecutive days; virus load was detected using the RT-qPCR method. Results are presented as mean ± SD. (**I**) Zebrafish were randomly selected from F_2_ generation-positive individuals, and the total protein was extracted after removing the head. The expression of scFv-1 protein was detected using the WB method. (**J**) The expression of the *scFv-1* gene in zebrafish (Tg group, 2 months old) heart, liver, brain, spleen, kidney, gill, intestine, and muscle tissues (*n* = 3). (**K**) The survival rate of zebrafish (2 months old) after SVCV infection within 14 days; the survival curve was calculated by GraphPad Prism software. (**L**) The virus titer analysis of zebrafish infected with SVCV (*n* = 5). Data are presented as mean ± SD. ***P < 0.001. (**M**) Analysis of pathological sections of the liver and spleen of zebrafish infected with SVCV. (**N**) Heat map display of cytokine and chemokine analysis after zebrafish infection with SVCV. (**O**) Neutralization of SVCV by scFv-1 protein expressed *in vivo* using immunohistochemical analysis.

In view of the superior neutralization properties of the scFv proteins, the Tol2 transposons donor containing the *mCherry* and *scFv-1* genes was constructed and injected into zebrafish embryos together with the Tol2 transposase mRNA (Fig. 5A). The fertilized eggs after microinjection were cultured and hatched, and then the surviving individuals were observed under a stereo-fluorescence microscope. The positive individuals showed red fluorescence in the eyes (Fig. 5B), and the positive rate of surviving embryos was 60%. Subsequently, the selected positive individuals were cultured separately until sexual maturity, and then test-crossed with wild-type (WT) zebrafish to obtain F_1_ individuals. After being cultured to sexual maturity, 10 individuals with strong fluorescence expression were selected. Then, the screened positive F_1_ individuals were numbered and cultured separately. After sexual maturity, they were hybridized with wild-type zebrafish to obtain F_2_ individuals. The presence of the *scFv-1* gene was verified in randomly selected positive individuals of the F2 generation (Fig. 5C).

To assess the neutralization ability of individuals expressing scFv-1, positive F_2_ individuals were selected for SVCV challenge. After bathing with SVCV, the survival rate of the progeny increased by 30% in most family lines (Fig. 5D). Then, the family line 3 (Tg group) with relatively stable and high survival rate was selected for further analysis. The survival rate of this line was increased by 34.6% after SVCV infection compared with that of the wild-type group (Fig. 5E). In order to further analyze the anti-viral effect, we examined the viral loads of the WT group and the Tg group after SVCV infection. As shown in Fig. 5F, the viral load of the Tg group was reduced 54-fold compared with that of the WT group on the fifth day after the viral infection. In addition, it was found that the viral load of the Tg group was significantly higher than that of the WT group in the dying zebrafish (Fig. 5G), indicating that scFv-1 enhanced the tolerance of zebrafish to SVCV. Meanwhile, by detecting the SVCV content in the aquaculture water environment, we found that the zebrafish with gene therapy infected with SVCV discharged less virus (Fig. 5H), indicating that scFv-1 can neutralize SVCV effectively *in vivo* and reduce the amount of SVCV discharged into the environment, thus effectively mitigating the horizontal transmission of SVCV. In addition, scFv-1 expression in the zebrafish after gene therapy (Tg group) was confirmed by Western blot assay (Fig. 5I).

Furthermore, to verify the longevity of scFv-1 expression, positive zebrafish individuals were cultured until the age of 2 months. Zebrafish individuals were selected randomly for PCR detection, and it was found the *scFv-1* gene was expressed in the heart, liver, brain, spleen, kidney, gill, intestine, and muscle, especially in gill tissue, with the highest expression level (Fig. 5J; Fig. S14). Then, the zebrafish were infected with SVCV, and the survival rate of the Tg group increased by 41.94% compared with that of the WT group (Fig. 5K). Similarly, the viral titer in the SVCV-infected zebrafish of the Tg group decreased from 10^6.5^ TCID_50_/mL to 10^4^ TCID_50_/mL (Fig. 5L). Simultaneously, pathological analysis of zebrafish liver and spleen showed that tissue damage caused by the SVCV infection was significantly alleviated in the Tg group (Fig. 5M). Meanwhile, the inflammatory response in the Tg group after SVCV infection was significantly attenuated through the analysis of cytokines and chemokines in the spleen (Fig. 5N; Fig. S15). To further confirm the role of scFv-1 expressed *in vivo* for neutralizing the SVCV, we collected the liver and spleen tissues from SVCV-infected zebrafish for immunofluorescence detection. The fluorescence signals generated by SVCV and scFv-1 were found overlapped. In addition, the fluorescence signal generated by SVCV in the Tg group was significantly weaker than that in the WT group (Fig. 5O). These results indicated that the neutralizing gene therapy by delivery of the *scFv-1* gene is effective in neutralizing SVCV and increasing zebrafish to resist SVCV.

## DISCUSSION

The occurrence of frequent outbreaks of viral diseases represents a significant threat to human public health and the development of animal husbandry and fisheries. This is particularly the case in the context of emerging pathogens, such as SARS-CoV-2 (1). How to quickly obtain new, safe, and effective therapeutic drugs is necessary for the rapid prevention and treatment of the disease. Phage display technology (PDT) represents an emerging and highly efficient technique for gene expression screening and is usually applied for screening antigenic determinants of protein of interest, as well as intent antibodies (33–35). It is noteworthy that PDT has the advantage of a short preparation time and a large library capacity, which allows for the rapid screening of effective therapeutic antibodies against pathogens in immune-antibody libraries (36, 37). In this study, we used the purified SVCV to immunize rabbits by multiple booster immunizations and constructed the corresponding immune-antibody library. Two single-chain antibodies (scFv’s) with high affinity were obtained by biopanning using the phage display technology. Although a large number of antibodies have been produced and applied in aquaculture, there were few antibodies for disease prevention and control, and most of them were mainly used for disease detection (38–40). In our study, we further evaluated the neutralization performance of the selected scFv’s. The results showed the two scFv’s had excellent neutralization activity *in vitro*, which reduced the replication of SVCV effectively (Fig. 2). Encouragingly, it has been reported that scFv’s screened by phage display technology exhibit effective neutralizing ability against SARS-CoV-2 and respiratory syncytial virus (41, 42), indicating that screening neutralizing antibodies is of great potential for the prevention and treatment of viral diseases.

The neutralizing ability of antibodies *in vitro* effectively predicts and assesses their anti-viral efficacy *in vivo*, and antibody-mediated neutralization *in vitro* is increasingly understood to be mediated by a variety of mechanisms (1). Numerous studies have shown that antibodies can neutralize viruses, for example, by inhibiting the contact of viral particles with cells and inducing aggregation of viral particles (43–45). In this study, we found that scFv-1 had anti-viral activity both before and after adsorption of viral particles to cells (Fig. 2), and we also found that antibodies were able to neutralize viruses by preventing some adsorption of viral particles to cells and inhibiting the release of viral particles from cells (Fig. 3). Preliminary investigations indicate that antibody binding to the cell membrane may be a mechanism that prevents the release of the virus. However, the specific mechanism of neutralization remains to be further explored. Previous researchers have found that neutralizing antibodies have an effect on viruses such as Chikungunya virus and enterovirus A; i.e., neutralizing antibodies can act at several stages of the viral infection process, because the antibodies break down or change the conformation of the viral proteins by binding to the viruses and spatially blocking the attachment to the cells (46–48). Homology modeling techniques can effectively predict the spatial structure of antibodies, and molecular docking technology can analyze the intermolecular forces (49). The combination of the two can provide fine molecular structure information and analyze key amino acids that play a role, which is necessary for analyzing how antibodies recognize antigens (50). The present study has revealed that amino acid positions Trp4, His31, Arg32, and Arg118 of the G3 segmentation of the G protein of SVCV are indispensable for antibody recognition, as determined through a combination of molecular docking and targeted mutagenesis. Similarly, Li et al. constructed two models of scFv against kitasamycin and analyzed the key amino acid sites recognized by kitasamycin through molecular docking, which provides guidance for the *in vitro* evolution of antibodies (51). It is irrefutable that the resolution of the natural structures of antigen-antibody facilitates a superior comprehension of the mechanisms by which antibodies function. Regrettably, this study was unsuccessful in resolving the corresponding structures despite repeated attempts. Consequently, further investigation and resolution of this problem are essential in subsequent studies.

Traditionally, disease prevention and treatment have relied mainly on antibiotics, chemical drugs, and vaccines, but the resulting increase in drug resistance and even the potential risks to human health have raised serious concerns (52, 53). At the same time, it is irrefutable that single injections of pharmaceuticals and vaccines, while efficacious in the short term, are unable to provide enduring protection over the long term due to the metabolic processes that occur and the subsequent decline in vaccine titers over time. Although nanocarrier systems based on organic or inorganic materials can extend the metabolism time of a drug or vaccine to a certain extent (54, 55), it seems that lifelong protection may never be achieved. At present, gene therapy based on gene transfer has greatly improved this concern (56, 57). In the present study, the zebrafish with constitutively expressing neutralizing antibody were obtained by transporting neutralizing genes using the Tol2 transposable system. Further analysis showed that after SVCV infection, the survival rate of individuals with gene therapy was 34.6% higher than that of wild-type zebrafish after the SVCV infection, and the viral load was 54-fold lower than that of wild-type zebrafish (Fig. 5). Besides, the expression of the neutralization gene is long-lasting and persistent. In addition, the researchers demonstrated a strong anti-mastitis capacity by targeted integration of the gene with a targeted insertion of the innate inflammatory regulatory sequence into the promoter region of the lysozyme gene in dairy goats, which was persistently expressed (58). The above suggested that this may be a useful therapeutic option for a wide range of retroviruses, including HIV. In addition, it is worth noting that the insertion site of the *scFv-1* gene in this study is random, and whether the fixed-point insertion can enhance the anti-viral ability deserves further study.

In summary, in this study, a single-chain antibody with good neutralizing activity was obtained from the phage display antibody library constructed based on SVCV immunization of New Zealand white rabbits, which can block multiple stages of viral replication and thus play a neutralizing role, and can be continuously expressed for a long period of time after being transported to the body by transposon to play a better neutralizing role. Theoretically, this study provides a certain reference for neutralizing gene screening and gene therapy, as well as a method for the prevention and treatment of unknown pathogens.

## MATERIALS AND METHODS

### Cells and virus

EPC cells, kindly provided by Prof. Ling-Bing Zeng from Yangtze River Fishes Research Institute (Wuhan, Hubei, China), were cultured using M199 medium (Hyclone, USA) containing 10% fetal bovine serum (FBS) (ZETA LIFE, USA) and placed in a humidified atmosphere at 25°C containing 5% carbon dioxide. SVCV (strain 0504), kindly provided by Prof. Qiang Li from Dalian Ocean University (Dalian, China), was amplified in EPC cells (59).

### Rabbit immunization

Healthy young New Zealand white rabbits (2 months) were immunized by four rounds of subcutaneous injections with purified SVCV as the immunogen. At the first round of immunization, after collection of blood from the ear vein (negative serum), 600 µL of purified SVCV mixed with 600 µL of Freund’s complete adjuvant was emulsified and administered to the rabbit via subcutaneous multi-point injection. The second, third and fourth immunizations were injected on days 21, 35, and 49, respectively, using the same methods as the first injection, with Freund’s complete adjuvant replaced by Freund’s incomplete adjuvant. Sera of days 7, 14, 28, 42, and 56 were collected separately, and the antibody titer of the sera was identified by ELISA with SVCV being coated as an antigen and horseradish peroxidase-conjugated goat anti-rabbit IgG Mab being used as a secondary antibody. After 2 days, spleen tissue was collected for constructing the SVCV-immunized rabbit scFv library (60). Furthermore, in order to detect the affinity of the positive serum with the SVCV, the infected cells with SVCV were fixed with 4% paraformaldehyde, then sequentially incubated with BSA, positive or negative serum, and FITC-goat anti-rabbit IgG (Proteintech, Inc, USA). Finally, the cells were observed under a fluorescence microscope.

### IF assay

The EPC cells growing into monolayers were incubated with SVCV (10^3^ TCID_50_/mL) for 1 h at 25°C, then washed and cultured for another 24 h. After that, the EPC cells were washed with PBS and fixed with 4% paraformaldehyde, then sequentially incubated with BSA, corresponding primary antibodies, and FITC-goat anti-rabbit IgG (Proteintech). Subsequently, the cells were stained with DAPI and observed under confocal microscopy (Andorra, UK).

### Serum neutralization of SVCV

The serum samples were diluted with serum-free M199 medium to set up a series of concentration gradients ranging from 1:20 to 1:640. The diluted serum or M199 (control) was incubated with SVCV (10^3^ TCID_50_/mL) at 25°C for 1 h, then the mixture was added to the pre-cultured monolayer EPC cells (100 µL/well) for 72 h. Then, the cell viability was detected by MTT assay (49). Besides, the indirect immunofluorescence was also used to assess the neutralizing effect of serum on SVCV.

### Construction, enrichment, and screening of anti-SVCV phage display scFv library

To obtain the phage display scFv library, previous research methods were used with some modifications (61, 62). In brief, the spleen tissue of SVCV-immunized rabbit was used to extract total RNA using Trizol reagent (Invitrogen, USA) in accordance with the product operation manual. Then, the RNA was used as the template for reverse transcription to cDNA using the HiScript Q RT SuperMix for qPCR kit (Vazyme, China) according to the product operation manual. The resulting cDNA was then used to amplify the variable regions of the heavy-chain and light-chain (V_H_ and V_L_) genes, using specific primers listed in Table S1. These amplified fragments were subsequently spliced together to form the whole scFv gene using SOE-PCR. Then, the scFv genes were ligated into pCANTAB 5E phagemid vectors and then transformed into *E. coli* TG1 electro-competent cells to express phage display scFv, which constructed the SVCV-immunized rabbit phage display scFv library. The size and diversity of the library were evaluated by coating mixed transformants on 2 × YT solid medium and randomly selected single colony sequences, respectively. Subsequently, M13K07 auxiliary phages were added for library amplification, and the titers of the phage libraries were calculated. Then, the enrichment and screening of phage scFv particles were performed (63). Positive identification of phages after each round of biopanning was carried out to ensure the selection was correct. After the fifth round of enrichment, the positive clones were selected to obtain the phage supernatant and then screened for scFv with high binding capacity by ELISA (the prepared helper phage was used as the negative control, while the PBS buffer was used as the blank control). Meanwhile, 10 positive clones with higher antibody titers were selected then sent to Sangon Biotech (Shanghai, China) for sequencing, and the obtained sequences were compared and analyzed for subsequent experiments.

### Anti-SVCV scFv expression, purification, and verification

The gene of positive anti-SVCV scFv colons were subcloned into pET-32a vectors then transformed into *E. coli* BL21 (DE3). Subsequently, the *E. coli* spp. containing recombinant plasmids were spread on Luria-Bertani (LB) solid medium (containing 100-µg/mL ampicillin), and the single colonies were randomly selected to grow in LB liquid medium (containing 100-µg/mL ampicillin) to logarithmic phase, which was identified by colony PCR and DNA sequencing. The positive inoculum was inoculated into the LB medium containing ampicillin resistance and left to grow to the logarithmic phase (OD_600_ value of 0.5) for induced expression of proteins using IPTG (isopropyl-beta-D-thiogalactopyranoside, final concentration of 1.0 mM). This was followed by sonication and denaturation of the fragmented inclusion body proteins with 8-M urea, followed by purification of the His-tagged proteins. Purified scFv proteins were refolded by gradient urea solution, dialyzed, and vacuum freeze-dried. To exclude the effect of endotoxin, a negative anti-SVCV scFv strain was purified in the same way. The dried antibody proteins were then validated by SDS-PAGE and Western-blot analysis. In addition, the affinity of the scFv’s to the virus was detected using ELISA and indirect immunofluorescence assay.

### BLI assay

BLI was used to quantify the binding capacity of scFv’s to SVCV G protein. Briefly, 10 mg/mL of biotinylated SVCV G protein super streptavidin biosensor was applied to the needle, which was then submerged in running buffers containing varying concentrations of scFv’s. Following each round of binding and dissociation, the sensor was subjected to a wash with assay buffer in order to remove any non-specifically bound proteins and establish a baseline. The raw kinetic data were generated using data acquisition software (ForteBio). The binding/dissociation rate constants (kon/koff) were calculated and the affinity (KD) was determined using data analysis software (ForteBio) with double reference subtraction.

### *In vitro* neutralization assays

The scFv-1 and scFv-2 were pre-incubated with SVCV (10^3^ TCID_50_/mL) for 1 h at 25°C based on the safe concentration. Toxicity assays were performed using Cell Counting Kit-8 (Beyotime, China). Then, the antibody-virus mixture was added to co-incubation culture with EPC cells growing to monolayer in 96-well plates or 6-well plates for 48 h. Subsequently, the cytopathic effect was observed, and the cell viability was detected by MTT assay to preliminary evaluate the neutralizing effect of scFv protein on SVCV. Meanwhile, the virus titer was determined by collecting the supernatant in a six-well plate according to Reed and Muench (64). To further detect the neutralization activity of scFv, the neutralization assays of scFv-1 and scFv-2 at different concentrations (1, 10 and 100 µg/ml) were carried out. Briefly, scFv-1 and scFv-2 were pre-incubated with SVCV (10^3^TCID_50_/mL) for 1 h at 25°C. Then, the antibody-virus mixture was added to co-incubation culture with EPC cells growing to monolayer in 6-well plates for 1 h. Followed the cells were washed with PBS for three times and culture for 48 h at 25°C. Subsequently, the viral RNA was extracted and purified for RT-qPCR detection.

### Determination of the growth curve of SVCV

The purified scFv protein with a final concentration of 100 µg/mL was pre-incubated with SVCV (10^3^TCID_50_/mL) for 1 h at 25°C, and the same concentration of BSA was used as the controls. When the EPC cells inoculated into 6-well plates grew to monolayer, the antibody-virus mixture was added to co-incubation culture, and the supernatant was collected at 24, 36, 48, 60, 72 and 96 h, respectively. The supernatant was stored at −80°C and freeze-thawed for three times, then TCID_50_ was determined. The values at different time points were used to reflect the growth curve of SVCV.

### Immunofluorescence neutralization assays

The scFv (the concentration of 100 mg/L) was pre-incubated with SVCV (10^3^TCID_50_/mL) for 1 h at 25°C. Subsequently, the antibody-virus mixture was added to the monolayer of EPC cells and cultured for 48 h, followed by fluorescence intensity using IF assay. Similarly, the cells were washed three times with PBS and stained with Hoechst 33258, YO-PRO-1, and PI (Beyotime), and observed using live cell imaging system to detect apoptosis (Cytation 5; BioTek, USA).

### Pre- and post-attachment neutralization assays

For pre-attachment neutralization assay, scFv’s (concentrations of 0.1, 1.0, 10.0, 100.0, and 200.0 mg/L) were pre-incubated with SVCV (10^4^ TCID_50_/mL) for 1 h at 4°C. Then, the antibody-virus mixture was added to the pre-cooled EPC cells and incubated for 1 h at 4°C. Then, the cells were washed with M199 and cultured for 48 h at 25°C. After that, the cell viability was detected by MTT assay. In addition, the viral RNA was extracted and purified for RT-qPCR detection. For post-attachment neutralization assay, EPC cells were co-incubated with SVCV (10^4^TCID_50_/mL) for 1 h at 4°C. The cells were washed with M199 medium three times, followed by the addition of scFv (concentrations of 0.1, 1.0, 10.0, 100.0, and 200.0 mg/L) for 1 h at 4°C. The medium was then replaced with M199 containing 2% FBS for 48 h at 25°C with 5% CO_2._ After that, the cell viability was detected by MTT assay. In addition, the viral RNA was extracted and purified for RT-qPCR detection.

### Attachment inhibition assays

The scFv (concentrations of 1, 10, and 100 mg/L) was pre-incubated with SVCV (10^4^TCID_50_/mL) for 1 h at 4°C, and then the antibody-virus mixture was added to the precooled EPC cells that grew to a monolayer and incubated for 2 h at 4°C. Subsequently, the cells were washed three times to remove the unabsorbed virus, and the viral RNA was extracted and purified for RT-qPCR detection. Furthermore, antibodies (at a concentration of 100 mg/L) were pre-mixed with SVCV, added to EPC cells that had been cultivated to form a monolayer on cell crawlers, and incubated at 4°C for 2 h. Subsequently, the cells were fixed with 4% paraformaldehyde and detected by IF assay (rabbit anti-SVCV polyclonal antibody serum as primary antibody and FITC-labeled goat anti-rabbit IgG as secondary antibody). Additionally, the cells were stained with Dil and DAPI, and the viral particles on the surface of the cells were observed by confocal microscopy after washing with PBS. The cells were prepared according to the aforementioned method, with the exception that only the viral particles were labeled, and the quantity of viral particles on the cell surface was determined by flow cytometry.

### Membrane fusion inhibition assay

The fusion detection of cell membrane was based on a previous study (65) and simply modified. Briefly, EPC cells that grew to monolayers were co-incubated with SVCV (10^2^ TCID_50_/mL) at 25°C. After co-incubation for 24 h, the cells were washed three times and then added to the cell fusion medium (M199 medium supplemented with 2% FBS, containing 20-mM 2-[4-(2-hydroxyethyl) piperazin-1-yl]ethanesulfonic acid and 20-mM morpholineethanesulfonic acid with or without scFv at pH 6.0. After incubation for 1 h at 25°C, 4% paraformaldehyde was added to fix the cells for 20 min, and then the cells were stained with crystalline violet staining solution (Beyotime) for 5 min. Afterward, the cells were washed with PBS and observed under an optical microscope.

### Virus egress inhibition assays

In 24-well plates, EPC cells were co-incubated with SVCV (10^3^ TCID_50_/mL) for 2 h at 25°C. Then, the cells were washed with M199 medium three times and continued to be cultured at 25°C with 5% CO_2._ The supernatant was collected at 2, 4, 6, 8, 10, 12, 18, and 24 h for virus detection. Subsequently, the same treatment was used. The difference was the washed cells were cultured in M199 containing scFv (concentrations of 1, 10, and 100 mg/L) for 2, 10, and 18 h at 25°C. In addition, the virus infection group and the BSA (the concentration of 100 mg/L) treatment group were set as controls. Afterward, the supernatant was collected, and the viral RNA was extracted and purified using EasyPure Viral DNA/RNA Kit (Transgen, China) for RT-qPCR detection. Subsequently, the scFv-1 was co-incubated with the EPC cell for 18 h at 25°C with 5% CO_2_, and the binding of the scFv-1 to the cell membrane was detected by direct immunofluorescence,

### Bioinformatics analysis of binding sites

In order to ascertain the site at which the scFv exerts its anti-viral function, bioinformatics and a WB assay were employed to analyze the binding sites. To exclude regions of non-specific binding, the SVCV-G protein was truncated for expression and subjected to a WB assay. Subsequently, the SVCV G protein truncated proteins and scFv’s were predicted and subjected to molecular docking using the AlphaFold3 Server. The amino acid residues of SVCV G3 that interact with scFv-1 were analyzed using PDBePISA. All protein three-dimensional structures and graphical illustrations were visualized and processed by PyMOL (Schrödinger, USA). The QuickMutation Site-Directed Mutagenesis Kit (Beyotime) was used to sequentially mutate the corresponding amino acid sites, and their binding was detected by WB in order to analyze the sites where the scFv-1 specifically recognizes the SVCV G protein.

### Evaluation of scFv-1 prophylactic and therapeutic experiments in zebrafish model

The healthy zebrafish were randomly divided into eight groups (60 zebrafish in each group) named groups T1, T2, T3, T4, P1, P2, P3, and P4. For prophylactic experiments, zebrafish in groups P2, P3, and P4 were intraperitoneally injected with 20-µL SVCV (10^3^ TCID_50_/mL), while the fish in group P4 were intraperitoneally injected with 20-µL scFv-1 (dose of 12.5 mg/kg) 6 h before challenge with SVCV. Besides, the fish in group P3 were intraperitoneally injected with 20-µL BSA (dose of 12.5 mg/kg), and the fish in group P1 were intraperitoneally injected with 20-µL PBS as control. For therapeutic experiments, zebrafish in groups T2, T3, and T4 were intraperitoneally injected with 20-µL SVCV (10^3^ TCID_50_/mL), and the zebrafish in group T1 were intraperitoneally injected with 20-µL PBS. The zebrafish in Group T2 were intraperitoneally injected with 20 µL scFv-1 (the dose of 10 mg/kg) 6 h after challenging with SVCV. Similarly, the zebrafish in group T3 were intraperitoneally injected with 20-µL BSA (the dose of 10 mg/kg) 6 h after SVCV infection. Then, the survival rate of each group was recorded during a 14-day experimental period.

Meanwhile, another set of experiments with the same treatment was carried out. On the third, fifth, and seventh days after injection, five zebrafish were collected from each group, and the liver, spleen, and kidneys, respectively, were homogenized using Trizol to extract total RNA and reverse transcribe into cDNA. Then, qRT-PCR was detected using the CFX96 real-time PCR detection system (Bio-Rad, USA). The viral burden was evaluated based on an absolute quantitative standard curve. In addition, zebrafish were collected on the seventh day after infection for virus titer detection.

### Histopathological analysis

The liver and spleen tissues of the zebrafish were washed with PBS and stored in 4% paraformaldehyde. The samples were dehydrated with a concentration gradient of ethanol and treated with xylene, followed by paraffin embedding, sectioning, and staining with hematoxylin-eosin (H&E). Afterward, the pathological alterations in tissue slices were observed using an optical microscope. At the same time, zebrafish were injected intraperitoneally with scFv-1 (at a concentration of 5 mg/kg) every 2 days for three consecutive injections. After 14 days, zebrafish were randomly selected, and liver and spleen tissues were collected for histopathological observation to evaluate the safety of the scFv-1.

### Tracking the biodistribution of antibody in zebrafish

The FITC (Beyotime) and scFv-1 protein were dissolved in dimethyl sulfoxide (DMSO) and 0.1-M sodium carbonate buffer solution (pH 9.0), and the final concentrations were 1 and 2 mg/mL, respectively. Then, the FITC solution was added to the scFv-1 protein solution (the volume ratio of FITC solution and scFv-1 protein solution was 1:200) and reacted at 4°C for 8 h in the dark. After the completion of the reaction, the unreacted dye was removed by dialysis, and then the labeled scFv-1 was freeze-dried under vacuum. All operations were carried out in dim light. The zebrafish was anesthetized and injected intraperitoneally with labeled antibody (concentration of 10 mg/kg) and then collected at different times (0, 0.5, 1.0, 2.0, 4.0, 8.0, 12.0, 24.0, 48.0, 72.0, 96.0, 120.0, 144.0, and 168.0 h) after intraperitoneal injection for *in vivo* imaging to detect the fluorescence intensity. All images were processed using the living body imaging system AniView 100 (BLT, China), and the same fluorescence threshold was used for comparison between groups.

### Tol2 transposon system construction

The Tol2 transposon plasmid and Tol2 transposase plasmid used in this work were kindly provided by Prof. Hou-Peng Wang. The plasmid contains the smallest element of the Tol2 transposon and the red fluorescent protein (mCherry) expression cassette. The *scFv-1* gene fragment was amplified, double digested, transformed, and sequenced to obtain the plasmid pT2 (crybb-mCherry-SV40-CMV-scFv-1-SV40) for driving scFv-1 expression. The plasmid expressed both red fluorescent protein and scFv-1 protein, and the red fluorescent protein was only expressed in the eyes. Tol2 transposase mRNA was prepared by *in vitro* transcription using SP6 mMESSAGE mMACHINE Kit (Thermo, USA) after the tol2 transposase plasmid was linearized through restriction endonuclease *NotI*. At the same time, the mRNA was purified using the MEGAclear Kit (Thermo) and diluted to a final concentration of 200 ng/µL.

### Microinjection

The zebrafish (wild-type AB strain) were kindly provided by Prof. Hou-Peng Wang. After zebrafish reproduction, the mixture of Tol2 transposon plasmid and Tol2 transposase mRNA (50 and 200 ng/µL, respectively) was injected into one-cell stage zebrafish embryos (about 200). Then, the zebrafish embryos were observed under a fluorescence microscope after microinjection to identify individuals with red fluorescence (positive). These positive individuals were then cultured to sexual maturity and hybridized with the wild-type zebrafish. The F_1_ generation of positive individuals was selected using fluorescence microscopy from the offspring resulting from the hybridization. Simultaneously, the F_1_ generation individuals were hybridized with the wild-type zebrafish, and the F_2_ generation-positive individuals were screened using fluorescence microscopy. After growing to sexual maturity, the zebrafish was randomly selected to cut the tail fin, and the expression of the *scFv-1* gene was detected after RNA extraction.

### Evaluation of therapeutic effects in zebrafish expressing scFv-1

The zebrafish (2 weeks old) in the transgenic group (Tg group) and the WT group were infected with SVCV (10^7^ TCID_50_/mL) by immersion. Then, the survival rate of each group was recorded during a 14-day experimental period. Another set of experiments with the same treatment was also conducted. Five zebrafish were collected every day after infection to detect the viral load. In addition, the dying zebrafish (the Tg group and WT group), and the water samples were also collected for viral load testing. The zebrafish were homogenized with Trizol to extract total RNA, and the RNA from water samples was extracted with the EasyPure Viral RNA Kit, followed by reverse transcription of the RNA into cDNA. Then, qRT-PCR was performed using the CFX96 real-time PCR detection system (Bio-Rad). The viral load was evaluated based on an absolute quantitative standard curve. Meanwhile, zebrafish (excluding the effect of red fluorescent protein in the head) were randomly selected to extract total protein, and the expression of the scFv-1 protein was detected using Western blot assay. Then, the zebrafish (2 months old) from family 3 were randomly selected to collect heart, liver, brain, spleen, kidney, gills, intestine, and muscle tissues to detect the expression of scFv-1 gene mRNA in the tissues through RT-qPCR assay. Similarly, the zebrafish (2 months old) were infected with SVCV by intraperitoneal injection, and the survival rate was recorded during a 14-day experimental period. Besides, the zebrafish were collected on the seventh day after infection for virus titer detection. At the same time, the spleen tissues of the zebrafish (WT or Tg group) were randomly collected, and total RNA was extracted. Then, the relative expression levels of cytokines and chemokines were detected using RT-qPCR assay. The liver and spleen tissues of the zebrafish were stained with H&E, and the pathological changes of the tissue sections were observed using an optical microscope.

### Fluorescence detection of scFv-1 binding to SVCV *in vivo*

Randomly selected F_2_ generation-positive zebrafish individuals (2 months old) were infected with SVCV (10^3^ TCID_50_/mL) by intraperitoneal injection. Three days after infection with the virus, liver and spleen tissues were collected, and tissue samples were embedded, fixed, and prepared into frozen sections. Then, the tissues were sequentially incubated with BSA and FITC-goat anti-rabbit IgG (Proteintech) after antigen repair and tissue permeability. Subsequently, it was washed with PBS and co-incubated with the corresponding primary antibody (purified scFv-1 protein) and corallite 594-conjugated mouse anti-6× His (Proteintech) in sequence. Then, the slices were stained with DAPI and observe under a fluorescence microscope (Lecia DM6B; Lecia, Germany).

### Statistical analysis

The data obtained in this study were analyzed using the SPSS 26.0 software (SPSS Inc., USA). One-way analysis of variance and Tukey’s test were used to analyze the differences between the controls and treatments, where *P* < 0.05 is considered significant.

## Data Availability

All data are available within the article and its supplemental material.

## SUPPLEMENTAL MATERIAL

Supplemental material jvi.01497-24-s0001.docx Figures S1 to S15; Tables S1 to S4.

ASM does not own the copyrights to Supplemental Material that may be linked to, or accessed through, an article. The authors have granted ASM a non-exclusive, world-wide license to publish the Supplemental Material files. Please contact the corresponding author directly for reuse.
