# Supplementary material for: Single-chain antibody gene therapy strategy based on high-throughput screening triggers sustained antiviral activity in the body
Source: J Virol. 2024 Dec 23;99(1):e01497-24. doi: 10.1128/jvi.01497-24 (PMC11784017; doi:10.1128/jvi.01497-24)
Supplement: Supplemental material — Figures S1 to S15; Tables S1 to S4. [file jvi.01497-24-s0001.docx]

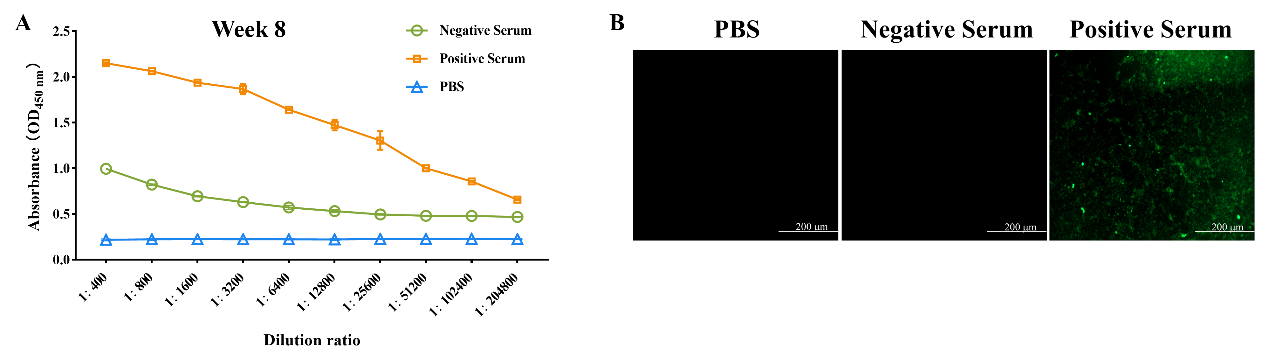


**Fig. S1** **Evaluation of affinity between serum and virus.** (A) The titer of polyclonal antibodies in the serum at the eighth week after immunization is detected by ELISA. (B) Detection of affinity between serum and virus by indirect immunofluorescence.


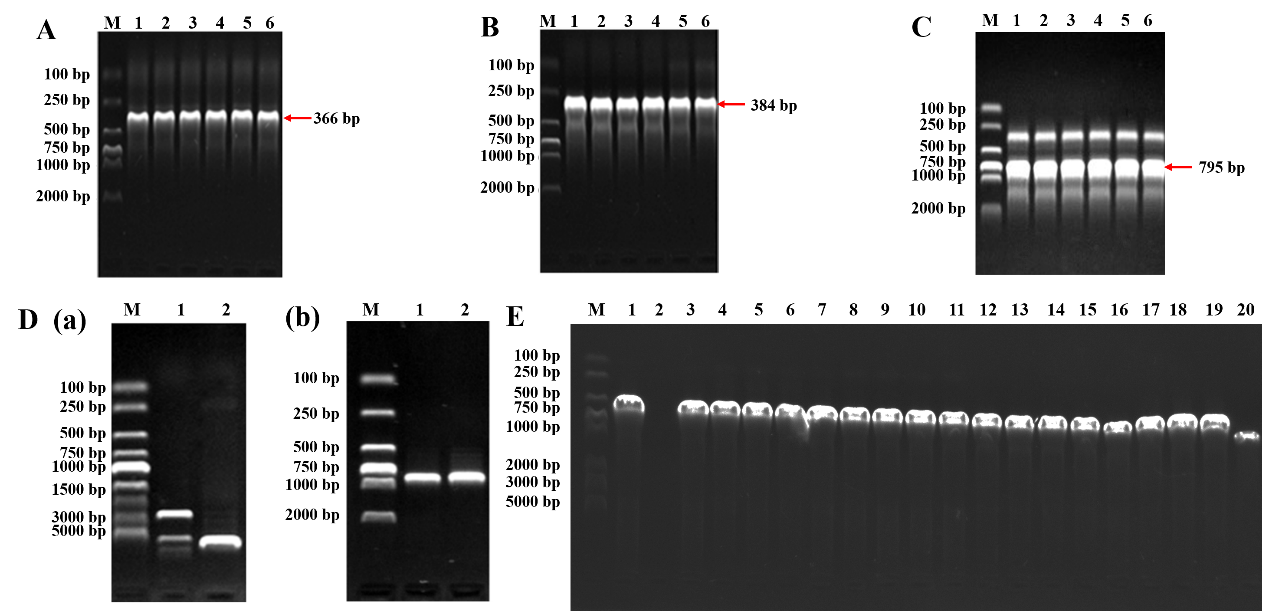


**Fig. S2** **Construction of phage display anti-SVCV antibody library**. (A) light chain gene amplification product. M: Marker, 1-6: V_L_ PCR products. (B) heavy chain gene amplification product. M: Marker, 1-6: V_H_ PCR products. (C) Nucleic acid electrophoresis pattern of scFv splicing products. M: Marker, 1-6: scFv PCR products. (D) Digested agarose gel electropherogram of vehicle (a) and scFv (b). (a) M: DNA maker; 1: non digested vector; 2: digested vector; (b) M: DNA maker; 1: non digested scFv; 2: digested scFv. (E) Verification of recombination rate of primary antibody library. M: Marker; 1-20: primary antibody library.


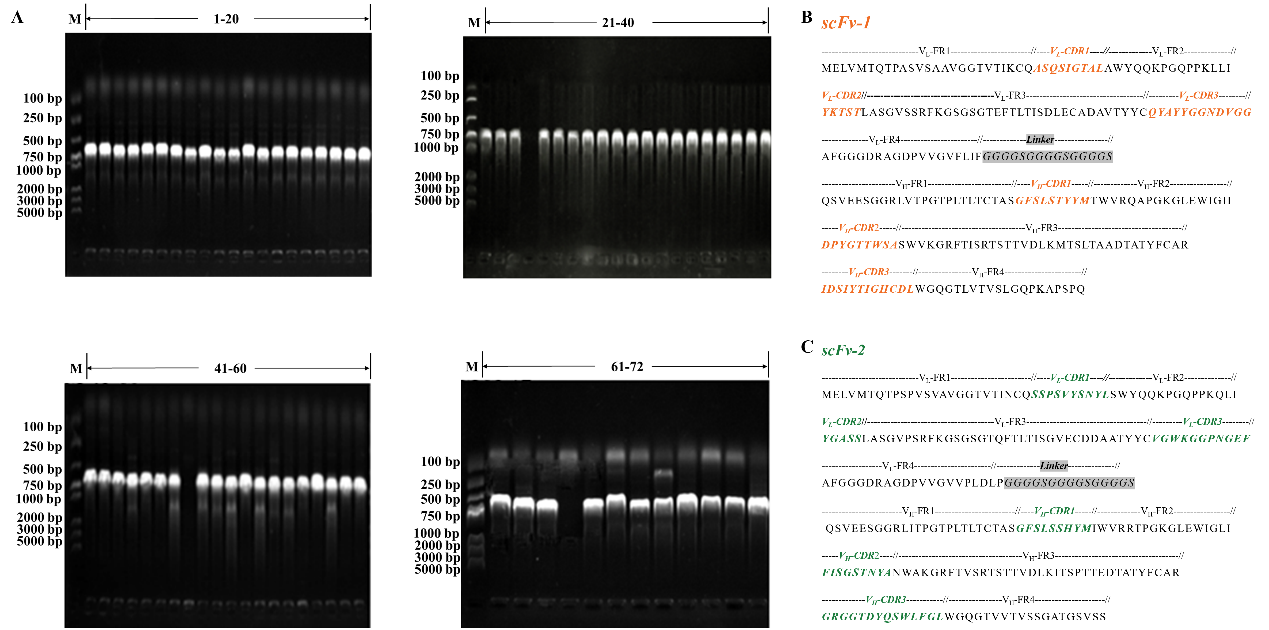


**Fig. S3** **Validation and sequence analysis of phage library.** (A) Identification of recombination rate of ultimate phage library. M: Marker; 1-72: plaque number. (B and C) Amino acid sequence analysis of the scFv-1 and the scFv-2.


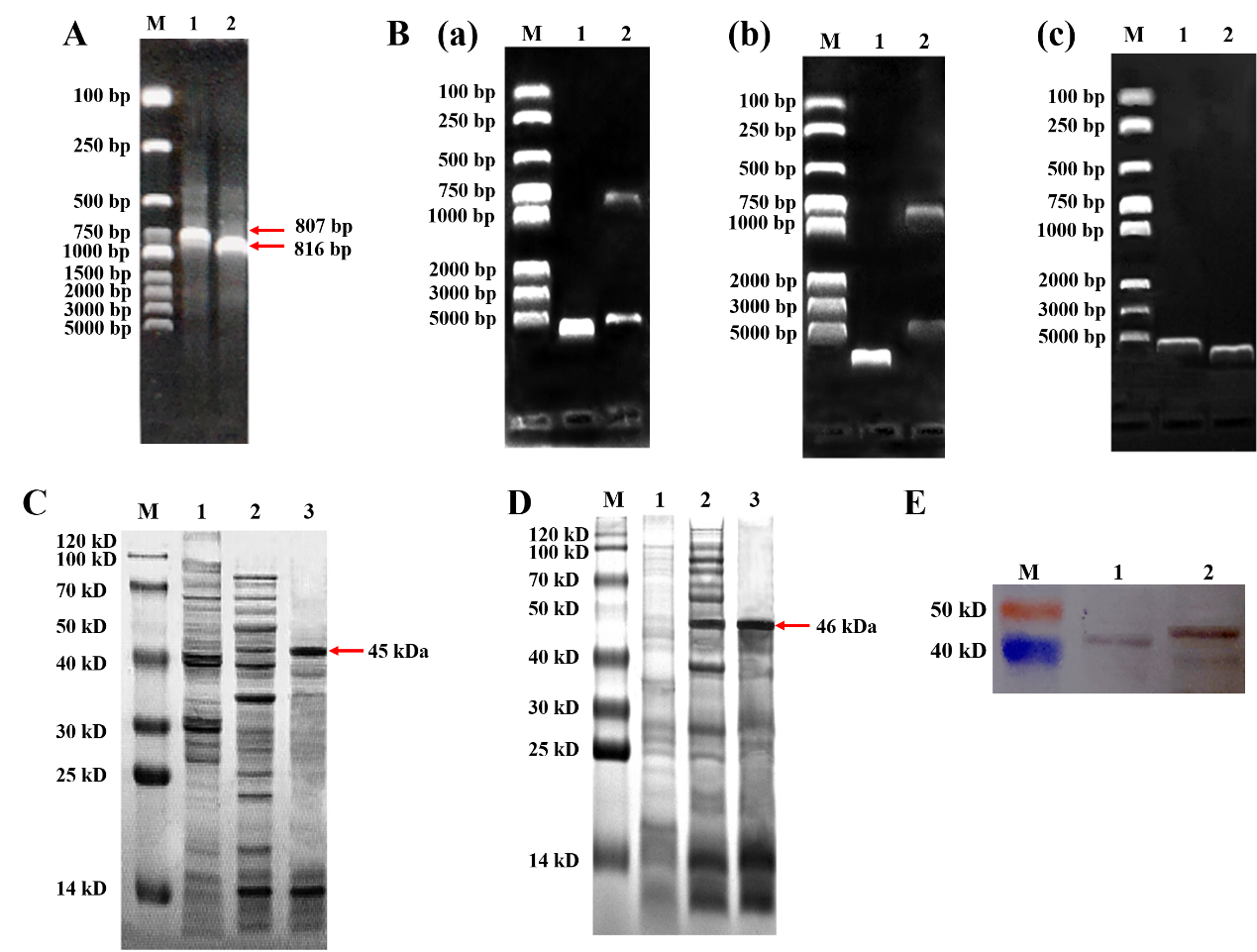


**Fig. S4** **Construction of expression vector and validation of scFvs.** (A) Amplification products of the *scFv* genes. M: Marker; 1: *scFv-1* gene amplified product; 2: *scFv-2* gene amplified product. (B) Verification of double enzyme digestion products of *scFv* genes and vector. (a) Double enzyme digestion products of recombinant cloned scFv-1 plasmid. M: Marker; 1 and 2: PMD-19T-scFv-1 recombinant plasmid; (b) Double enzyme digestion products of recombinant cloned scFv-2 plasmid. M: Marker; 1 and 2: PMD-19T-scFv-2 recombinant plasmid; (c) Double enzyme digestion products of pET-32a vector plasmid. M: Marker; 1 and 2: pET-32a vector plasmid. (C) SDS-PAGE analysis of scFv-1 protein expression. M: Maker; 1: the expression of pET-32a vector protein; 2: the expression of pET-32a-scFv-1 protein; 3: purified scFv-1 protein. (D) SDS-PAGE analysis of scFv-2 protein expression. M: Maker; 1: the expression of pET-32a vector protein; 2: the expression of pET-32a-scFv-2 protein; 3: purified scFv-2 protein. (E) WB validation of the scFv proteins. M: Maker; 1: purified scFv-1 protein; 2: purified scFv-2 protein.


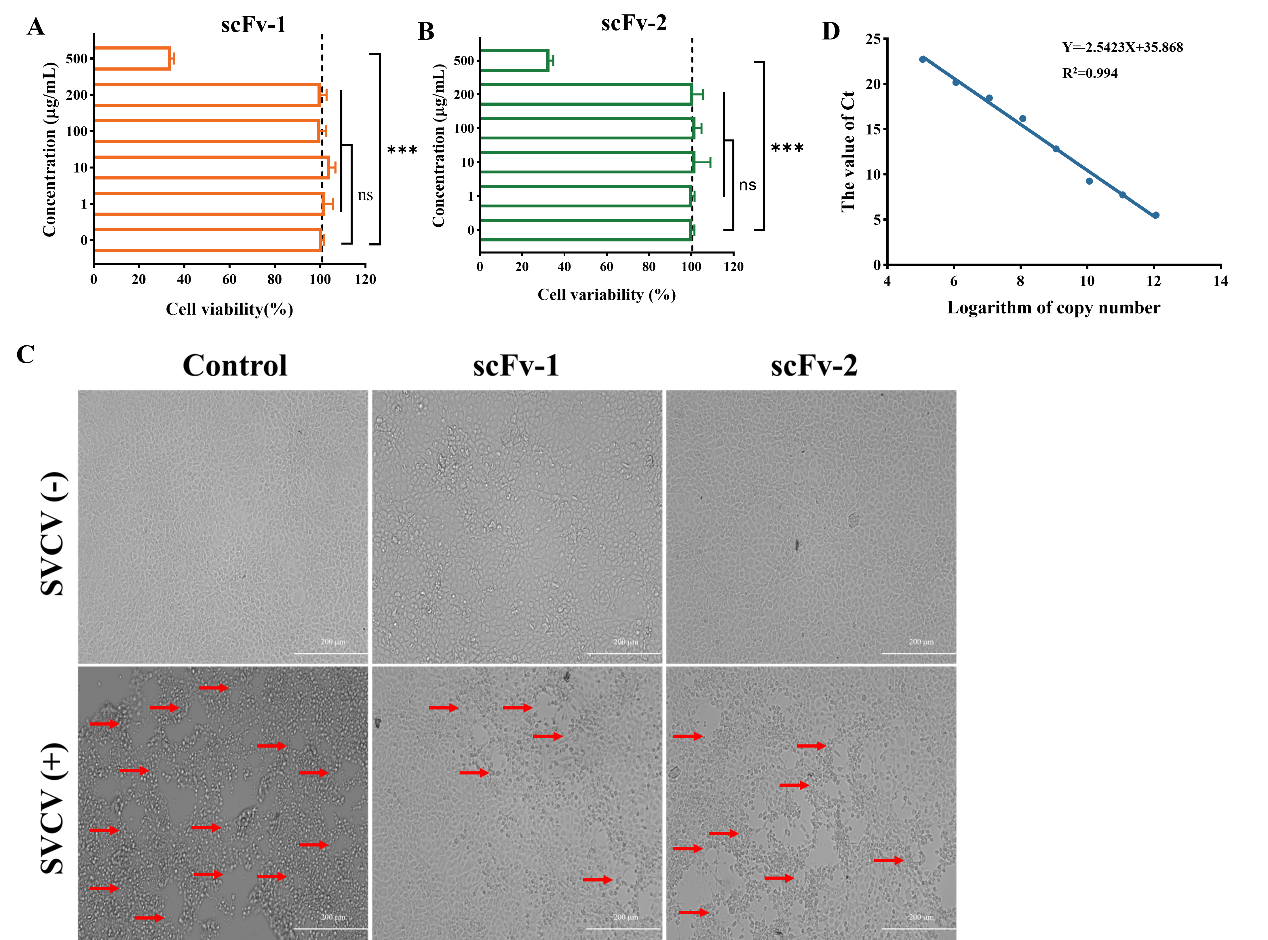


**Fig. S5** **Preliminary evaluation of the safety and neutralizing activity of single-chain antibodies.** The percentage of cytotoxicity of the scFv-1 (A) and the scFv-2 (B) in EPC cells. Toxicity was detected by MTT assay, and expressed as cell viability (%). Results were from a minimum of three replicates and values were presented as means ± SD. (C) Morphological observation of scFvs neutralize SVCV in EPC cells. (D) Standard curve of *SVCV-G* gene. A linear regression relationship between serial dilution plasmids and Ct values was formulated with a correlation coefficient (R^2^) of 0.994 and a slope of -2.5423.


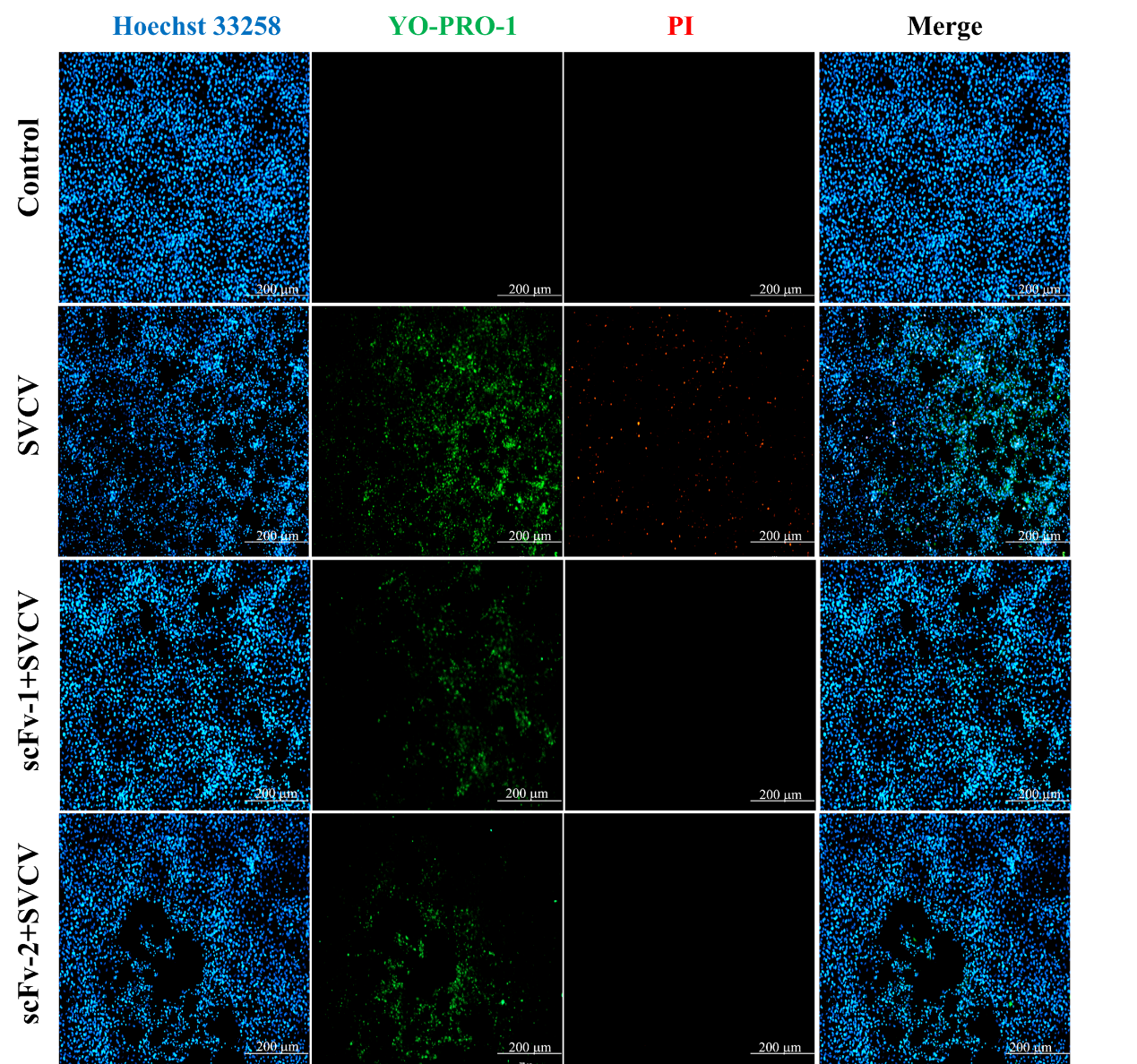


**Fig. S6** **The single-chain antibodies neutralize virus to improve cell apoptosis caused by SVCV.** The nucleus was stained with Hoechst 33258 (blue fluorescence), and the apoptotic cells and necrotic cells were stained with YO-PRO-1 (green fluorescence) and PI (red fluorescence), respectively. Scale: 200 µm.


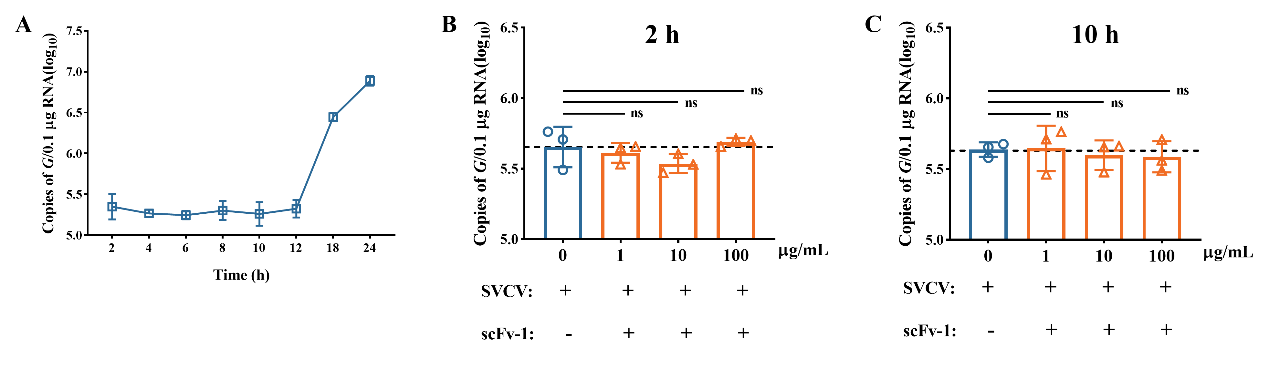


**Fig. S7** **The scFv-1 can not inhibit the release of the SVCV in the early stages**. (A) Virus content in supernatant at different time points after SVCV infection of EPC cells. After infecting EPC cells with SVCV for 2 h, the un-adsorbed virus was washed away and different concentrations of scFv-1 were added. The virus load in the supernatant was detected by RT-qPCR after adding scFv-1 for 2 h (B) and 10 h (C). Results are expressed as means ± SD.


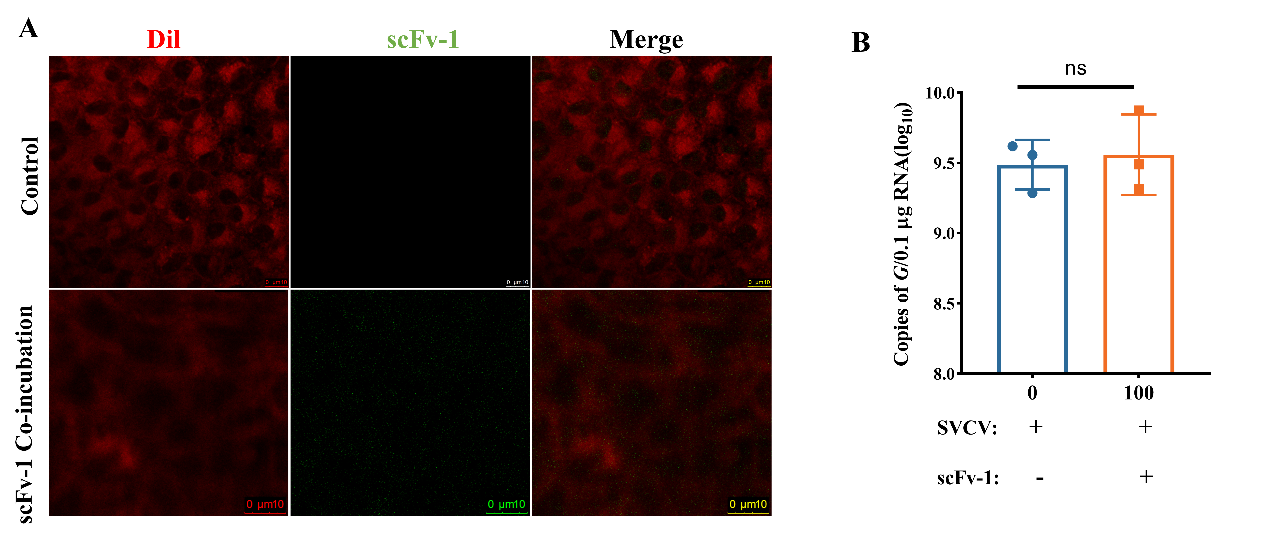


**Fig. S8 The preliminary investigation of the mechanisms by which scFv-1 inhibit viral exocytosis. (A)** The scFv-1 can bind to cell membranes to a certain extent after co-incubation with cells. Red: cell membrane; Green: scFv-1. **(B)** After infecting EPC cells with SVCV for 2 h, the un-adsorbed virus was washed away and different concentrations of scFv-1 were added. The virus load in the supernatant was detected by RT-qPCR after adding scFv-1 for 48 h. Results are expressed as means ± SD.


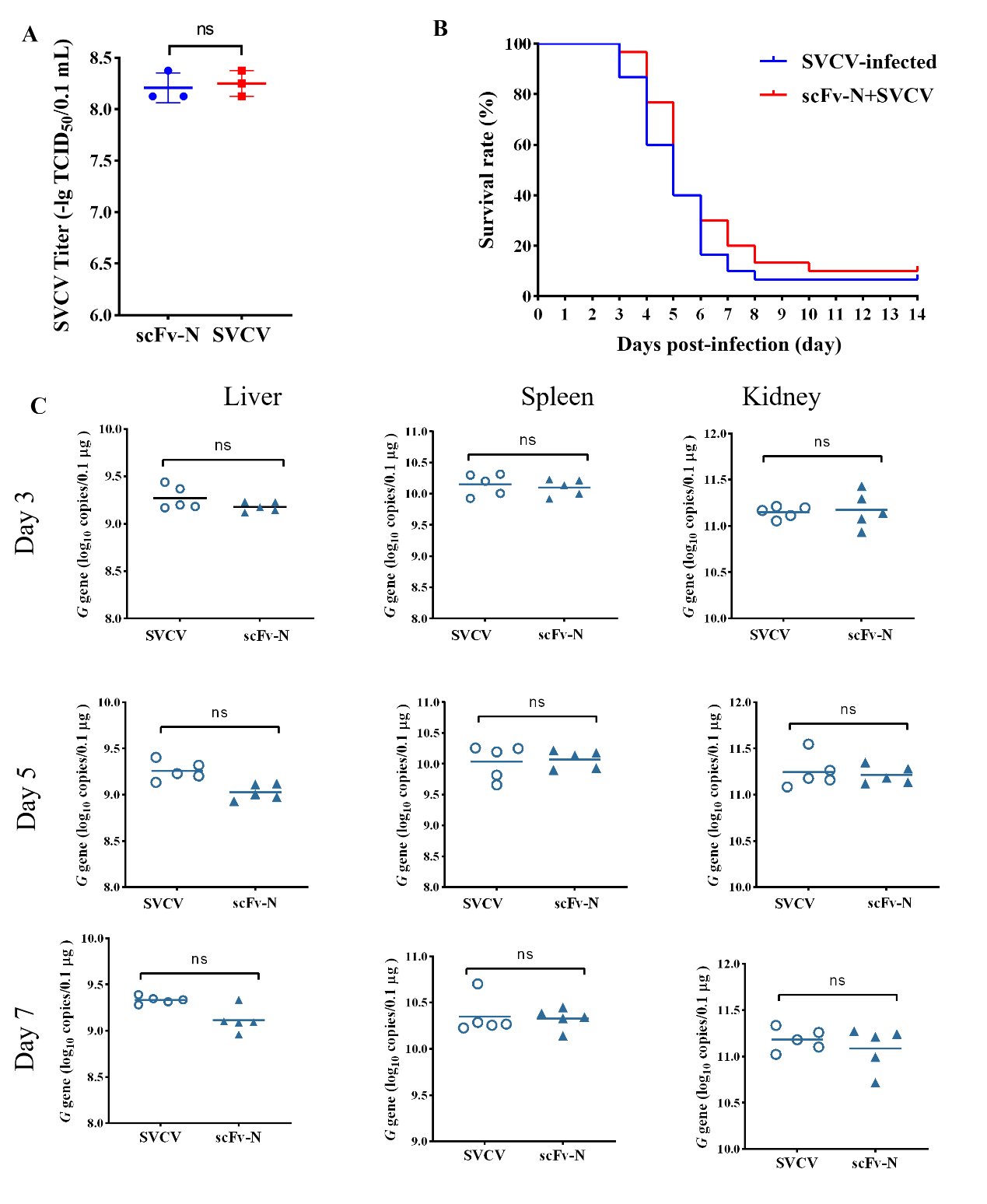


**Fig. S9** **Evaluation of therapeutic effect of the scFv-N. (A)** Titer analysis of virus after scFvs treatment in EPC cells. **(B)** Survival rate of SVCV infected fish within 14 days after scfv-N treatment, and the survival curve was calculated by GraphPad Prism software. **(C)** The viral load in the liver, spleen and kidney of SVCV-infected zebrafish on the days 3^rd^, 5^th^ and 7^th^ after scFv-N treatment, respectively.


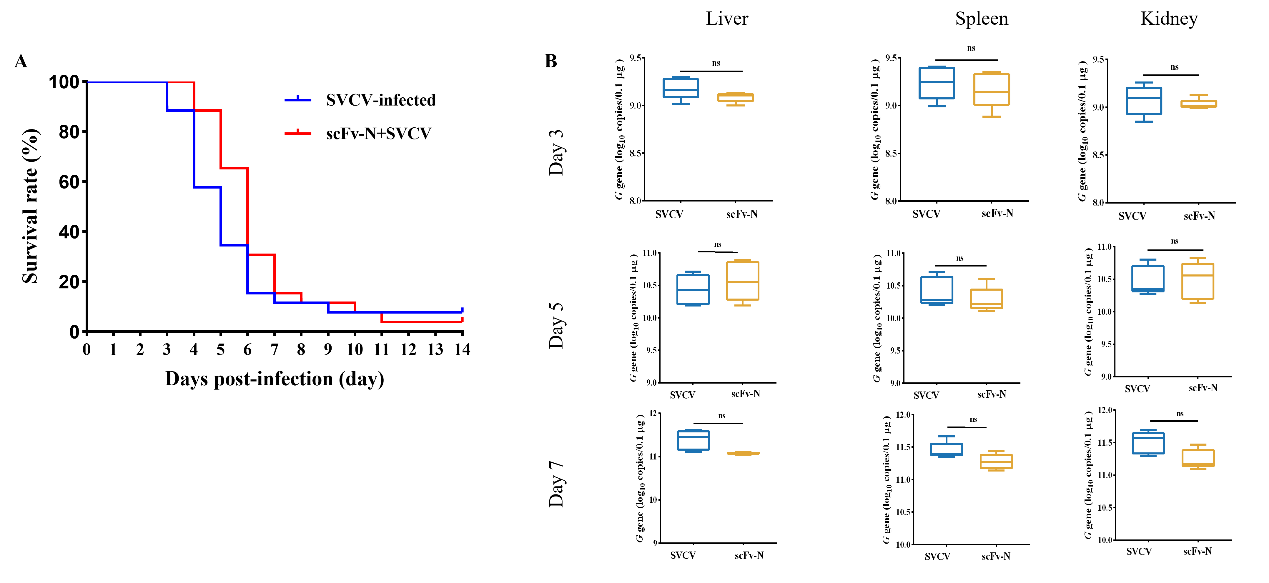


**Fig. S10** **Evaluation of preventive effect of the scFv-N. (A)** Survival rate of SVCV infected fish within 14 days after scfv-N pre-treatment, and the survival curve was calculated by GraphPad Prism software. **(B)** The viral load in the liver, spleen and kidney of SVCV-infected zebrafish on the days 3^rd^, 5^th^ and 7^th^ after scFv-N pre-treatment, respectively.


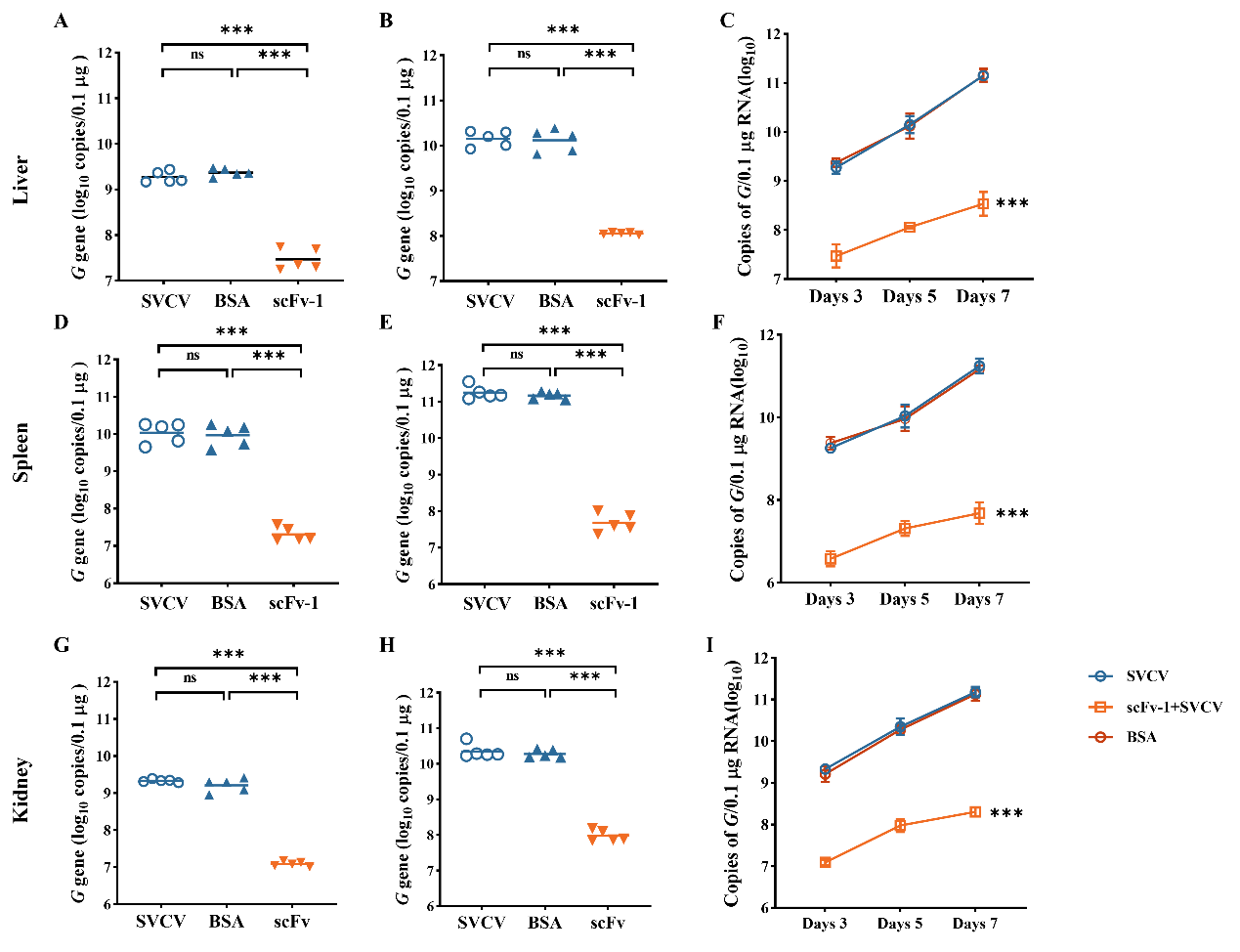


**Fig. S11** **Evaluation of therapeutic effect of the scFv-1.** The viral load was evaluated by RT-qPCR, and expressed as copies of SVCV *G* gene. Values are presented as mean ± SD. (A - B) The viral load in the liver of SVCV-infected zebrafish on the days 3^rd^ and 5^th^ after scFv-1 treatment, respectively. ***P* < 0.01, ****P* < 0.001. (D - E) The viral load in the spleen of SVCV-infected zebrafish on the days 3^rd^ and 5^th^ after scFv-1 treatment, respectively. ***P* < 0.01, ****P* < 0.001. (G - H) The viral load in the kidney of SVCV-infected zebrafish on the days 3^rd^ and 5^th^ after scFv-1 treatment, respectively. ***P* < 0.01, ****P* < 0.001. Growth curves of viral load in the liver (C), spleen (F) and kidney (I) of SVCV-infected zebrafish treated with scFv-1 were detected.


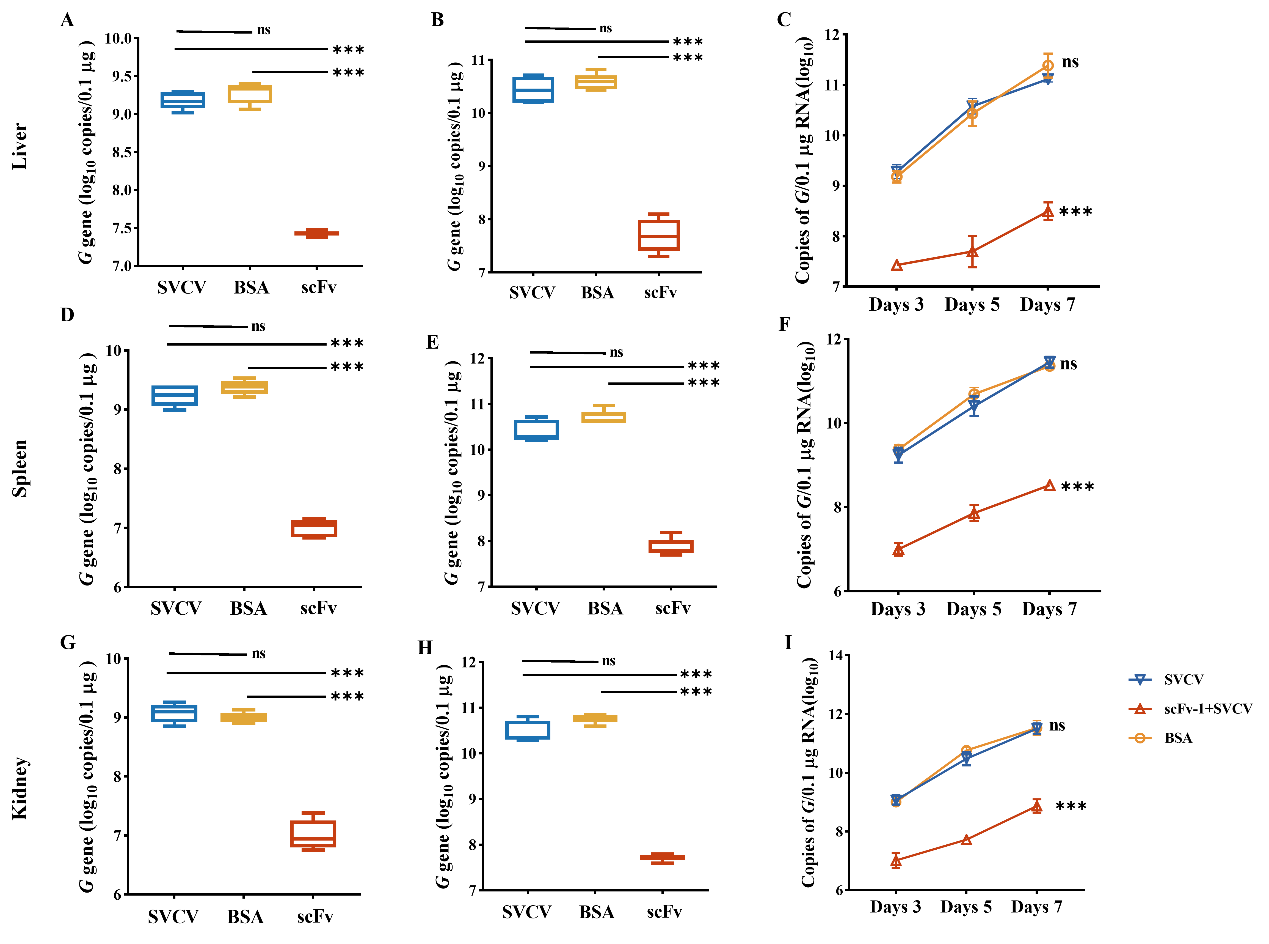


**Fig. S12** **Evaluation of preventive effect of the scFv-1.** The viral load was evaluated by RT-qPCR, and expressed as copies of SVCV *G* gene. Values are presented as mean ± SD. (A - B) The viral load in the liver of SVCV-infected zebrafish on the days 3^rd^ and 5^th^ after scFv-1 pre-treatment, respectively. ***P* < 0.01, ****P* < 0.001. (D - E) The viral load in the spleen of SVCV-infected zebrafish on the days 3^rd^ and 5^th^ after scFv-1 pre-treatment, respectively. ***P* < 0.01, ****P* < 0.001. (G - H) The viral load in the kidney of SVCV-infected zebrafish on the days 3^rd^ and 5^th^ after scFv-1 pre-treatment, respectively. ***P* < 0.01, ****P* < 0.001. Growth curves of viral load in the liver (C), spleen (F) and kidney (I) of SVCV-infected zebrafish pre-treated with scFv-1 were detected.


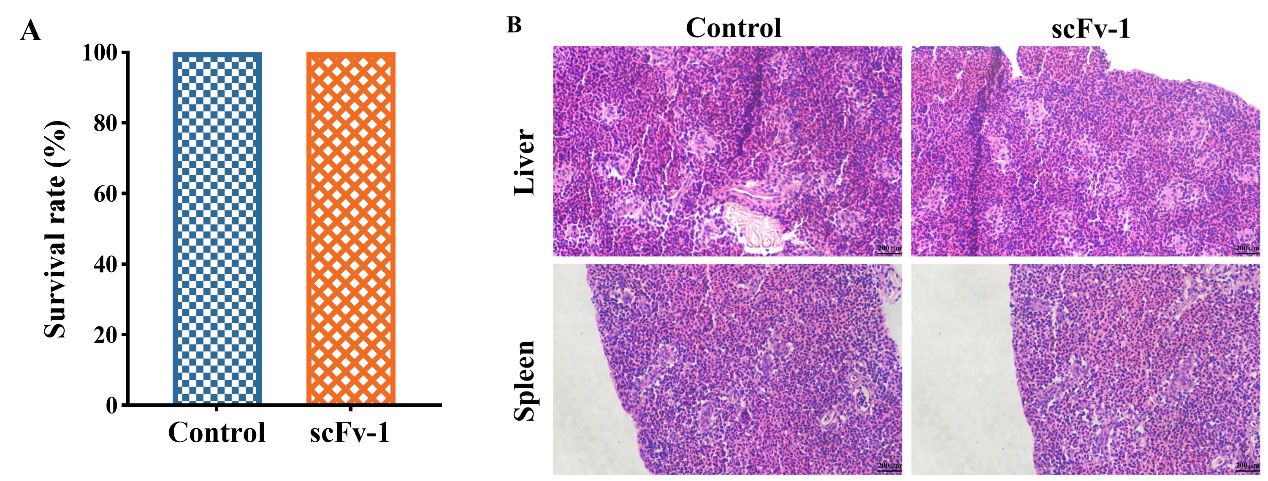


**Fig. S13** **Safety assessment of the scFv-1.** (A) Survival analysis after the scFv-1 treatment. (B) H&E staining was used to observe pathological changes in liver and spleen tissues after treated with scFv-1.


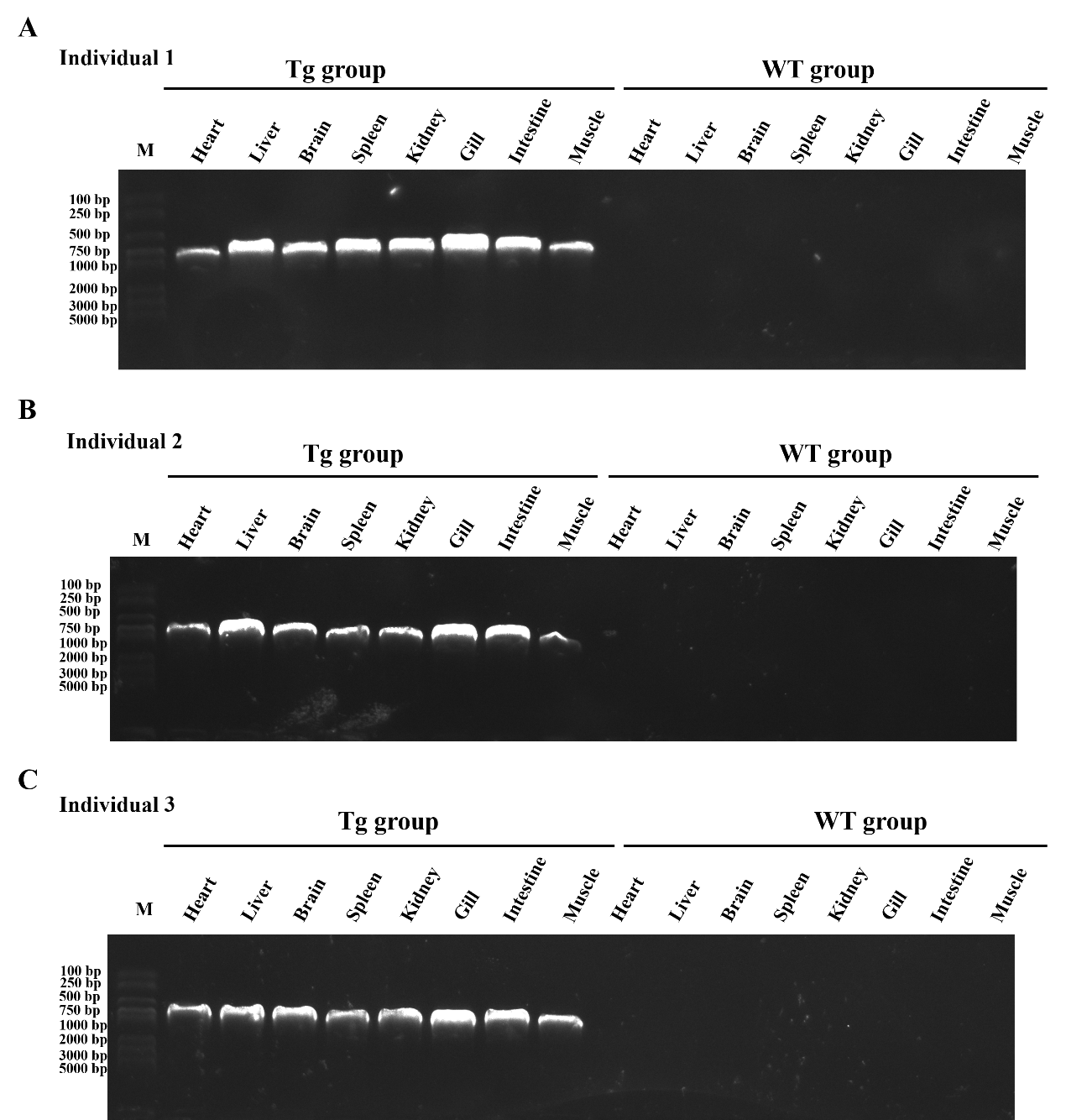


**Fig. S14** **The expression of scFv-1 in zebrafish.** (A-C) The zebrafish (two-month-old) were selected randomly from the WT and Tg group to collect the heart, liver, brain, spleen, kidney, gill, intestine and muscle to extract the total RNA to detect the expression of *scFv-1* gene.


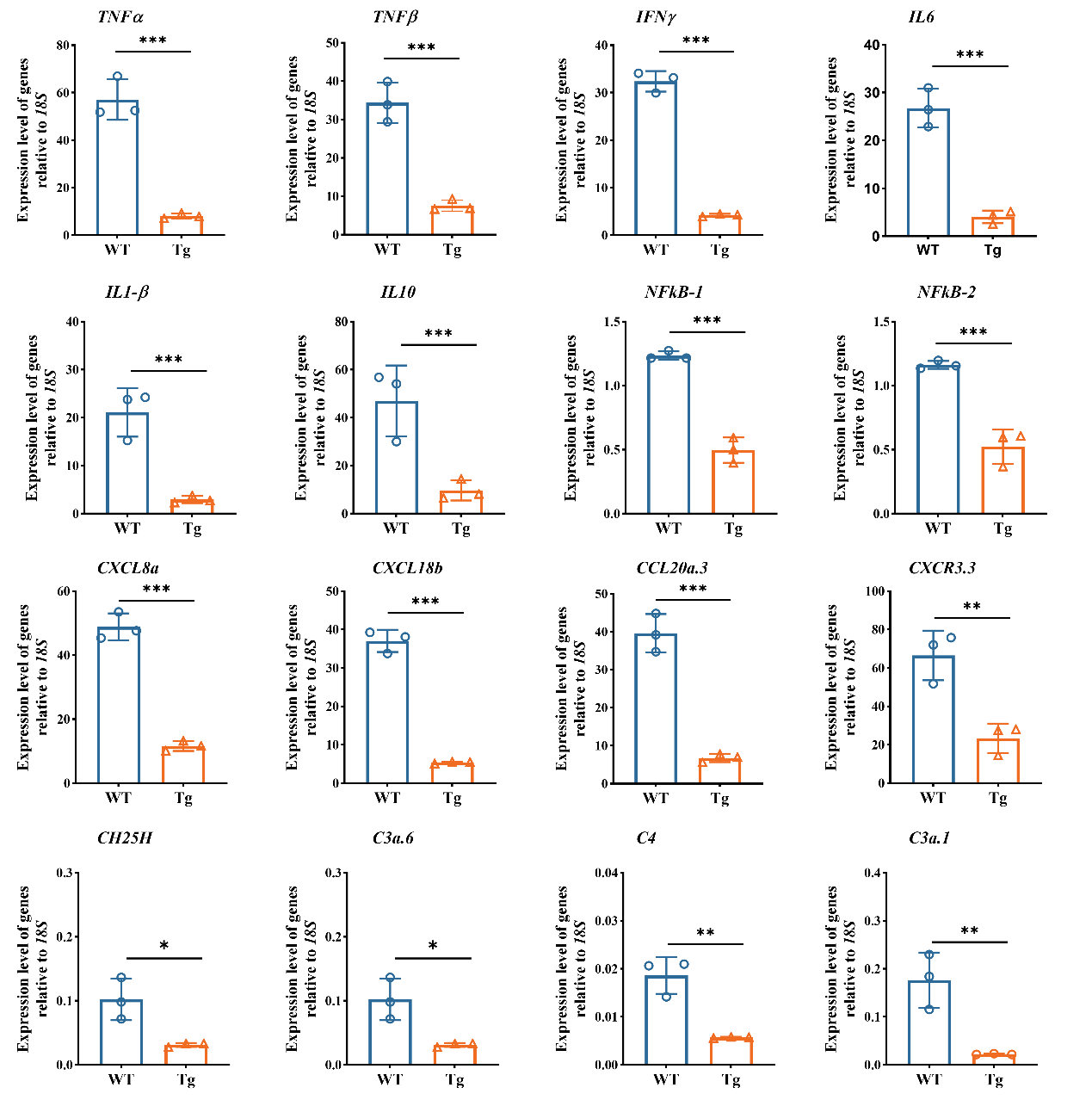


**Fig. S15** **Cytokines and chemokines levels in the spleen of SVCV-infected zebrafish.** The spleen tissues of zebrafish in the WT group and Tg group were collected on the 7^th^ day after SVCV infection, and the levels of cytokines and chemokines were detected using RT-qPCR assay. ***P* < 0.01, ****P* < 0.001.

Table S1 The primers used for construction of the SVCV immunized rabbit phage display scFv library

| Primer names | Primer sequences (from 5’ to 3’) |
| --- | --- |
| VL-F | GGGCCCAGCCGGCCATGGAGCTCGTGATGACCCAGACTCCA |
| VL-R | GGAAGATCTAGAGGAACACCCCCACCACCGGATCTCCAGCTCGGTCTCCTCCGCCGAAA |
| VH-F | CAGTCGGTGGAGGAGTCCGGG |
| VH-R | CCTGCGGCCGCCTGAGGAGACGGAGCCTTAGGTTGCCCCA |
| VL-Linker-R | TGAACCACCTCCACCGGAACCCCCACCACCGGAAGATCTAGAGGA |
| Linker-VH-F | GGTGGAGGTGGTTCAGGCGGAGGTGGCTCTCAGTCGGTGGAGGAGTCC |

Table S2 The primer sequence of recombinant plasmid construction

| Primer names | Primer sequences (from 5’ to 3’) |
| --- | --- |
| scFv-F | GGATCCATGGAGCTCGTGATGACC |
| scFv-R | CTCGAGCTGAGGAGACGGAGCCTT |

Table S3 Information of primers used for PCR and qPCR analysis

| Primer names | Primer sequences (from 5’ to 3’) | Application |
| --- | --- | --- |
| scFv1-F | AAGCTTGCCGCCACCATGGAGCTCGTGATGACC | PCR of scFv-1 |
| scFv1-R | GGATCCCTACTGAGGAGACGGAGCCT |  |
| scFv-1-F | TGCCGATGCTGTCACTTA | qPCR of scFv-1 |
| scFv-1-R | CACCGAAGATGAGGAACAC |  |

Table S4 The primer sequence of inflammatory cytokines and chemokines

| Primer names |  | Primer sequences (from 5’ to 3’) |
| --- | --- | --- |
| *TNFα-F* | NM-212859.2 | TACCGCTGGTGATAGTGTCC |
| *TNFα-R* |  | CTGGGTCTTATGGAGCGTGA |
| *TNFβ-F* | NM-001024447.1 | CTGGCATGTGATGAAGCCAA |
| *TNFβ-R* |  | AGTGAATGGCAGCCTTTGTG |
| *IFNγ-F* | NM-212864.1 | AATGACAGCGTGGATGAAGC |
| *IFNγ-R* |  | GCTCAAACAAAGCCTTTCGC |
| *IL-6-F* | XM-056480204.1 | TGACCTCAGTCCTGGTGAAC |
| *IL-6-R* |  | TCGATCATCACGCTGGAGAA |
| *IL1-β-F* | NM-212844.2 | TGGACTTCGCAGCACAAAATG |
| *IL1-β-R* |  | GTTCACTTCACGCTCTTGGATG |
| *IL-10-F* | NM-001020785.2 | TGGAGACCATTCTGCCAACA |
| *IL-10-R* |  | GCATTTCACCATATCCCGCT |
| *NFκB1-F* | XM-021481269.1 | AGGCCAAAGACACTGTTCGG |
| *NFκB1-R* |  | GGAAAGGTTGTGGGGTCCAT |
| *NFκB2-F* | NM-001001840.3 | AACAAGACGCAAGGAGCCCA |
| *NFκB2-R* |  | CTGTCTCTTGCACAAAGGGC |
| *CXCL8a-F* | XM-009306855.3 | GAAAGCCGACGCATTGGAAA |
| *CXCL8a-R* |  | TTAACCCATGGAGCAGAGGG |
| *CXCL18b-F* | NM-001115060.1 | CTCTCGTGAATCGTGCTCGC |
| *CXCL18b-R* |  | TGCAGTAATTGGCCCTGCTG |
| *CCL20a.3-F* | NM-056467903.1 | GAGTCGGATTTCAGCGTGTG |
| *CCL20a.3-R* |  | TCTTGCCTCCGTTTGTGTGG |
| *CXCR3.3-F* | XM-005173495.4 | GGAGGAGCAAGGTCCACTTC |
| *CXCR3.3-R* |  | AGAAACAAAGAGTCTCTCGGTCA |
| *CH25H-F* | NM-001008652.1 | GTCCTTCTGGATGCCCTGTC |
| *CH25H-R* |  | GGTTGTAGAGGGAGAGTGCG |
| *C3a.1-F* | NM-131242.1 | CGATGCAGGGCTGATGTTTG |
| *C3a.1-R* |  | TACTGACCAGCCAGTGTTGC |
| *C3a.6-F* | NM-001008582.4 | CCCGAGTGTGTCATCCCTTC |
| *C3a.6-R* |  | GTTCACACGTGTAGCCCAGT |
| *C4-F* | XM-021466464.1 | TGCGGTCTCAGTTTTGGACA |
| *C4-R* |  | ACCACCAGGGGTGAAATACG |
| *18S-F* | XM-030411820.1 | ACCACCCACAGAATCGAGAAA |
| *18S-R* |  | GCCTGCGGCTTAATTTGACT |
